# Coevolution of Lentiviral Vif with Host A3F and A3G: Insights from Computational Modelling and Ancestral Sequence Reconstruction

**DOI:** 10.3390/v17030393

**Published:** 2025-03-10

**Authors:** David Nicolas Giuseppe Huebert, Atefeh Ghorbani, Shaw Yick Brian Lam, Mani Larijani

**Affiliations:** 1 Immunology and Infectious Diseases Program, Division of Biomedical Sciences, Faculty of Medicine, Memorial University of Newfoundland, St. John’s, NL A1C 5S7, Canada; david_huebert@sfu.ca (D.N.G.H.); atefeh_ghorbani@sfu.ca (A.G.); 2 Structural Biology and Immunology Program, Department of Molecular Biology and Biochemistry, Faculty of Science, Simon Fraser University, Burnaby, BC V5A 1S6, Canada; brian_lam_2@sfu.ca

**Keywords:** Apobec3, Vif, lentiviruses, host–viral coevolution, viral antagonists, deamination, computational structure

## Abstract

The evolutionary arms race between host restriction factors and viral antagonists provides crucial insights into immune system evolution and viral adaptation. This study investigates the structural and evolutionary dynamics of the double-domain restriction factors A3F and A3G and their viral inhibitor, Vif, across diverse primate species. By constructing 3D structural homology models and integrating ancestral sequence reconstruction (ASR), we identified patterns of sequence diversity, structural conservation, and functional adaptation. Inactive CD1 (Catalytic Domain 1) domains displayed greater sequence diversity and more positive surface charges than active CD2 domains, aiding nucleotide chain binding and intersegmental transfer. Despite variability, the CD2 DNA-binding grooves remained structurally consistent with conserved residues maintaining critical functions. A3F and A3G diverged in loop 7’ interaction strategies, utilising distinct molecular interactions to facilitate their roles. Vif exhibited charge variation linked to host species, reflecting its coevolution with A3 proteins. These findings illuminate how structural adaptations and charge dynamics enable both restriction factors and their viral antagonists to adapt to selective pressures. Our results emphasize the importance of studying structural evolution in host–virus interactions, with implications for understanding immune defense mechanisms, zoonotic risks, and viral evolution. This work establishes a foundation for further exploration of restriction factor diversity and coevolution across species.

## 1. Introduction

Across species, the dynamic interactions between viruses and host organisms represent an ongoing evolutionary arms race, shaped by long periods of host–virus coevolution. This intricate relationship is particularly well studied in retroviruses where host restriction factors, such as those in primates, evolve to inhibit viral elements, while lentiviral proteins adapt to circumvent host defenses [1,2,3]. A prominent example of this interplay is the interaction between the host mutational enzyme apolipoprotein B mRNA editing enzyme catalytic polypeptide-like 3 (APOBEC3 or A3) and the lentiviral viral infectivity factor (Vif) [4]. APOBEC3 proteins act as restriction factors that induce mutations in viral genomes, while Vif counteracts their effects by targeting APOBEC3 proteins for degradation through the ubiquitination pathway. Lentiviruses are found in a wide range of animals, including ruminants, equines, felines, and primates, though notably absent in New World monkeys (NWMs) [5]. The zoonotic transmission of these viruses from one host species to another involves three critical steps: (1) exposure to the virus, (2) spillover into a new host species, and (3) establishment and spread within the new host population [6]. Successful transmission depends on the virus’s ability to recognize host cell receptors for entry and evade host innate restriction factors. Human immunodeficiency virus (HIV) exemplifies this process. HIV-1 originated from simian immunodeficiency virus (SIV) strains found in chimpanzees (*Pan troglodytes*) and gorillas (*Gorilla gorilla*), while HIV-2 originated from SIV strains infecting sooty mangabeys (*Cercocebus atys*). While SIV has crossed into humans on at least 11 occasions, only since the 1970s has it given rise to the epidemic forms of HIV [7,8,9,10,11]. In contrast, SIV has been infecting great apes (GAs) and Old World monkeys (OWMs) for at least 30,000 years, resulting in diverse clinical outcomes depending on the host species [12]. This evolutionary backdrop provides a unique framework to study the cellular and molecular interactions that govern host–virus dynamics.

HIV primarily infects immune cells, including T helper cells, macrophages, and certain dendritic cells [13,14,15,16,17,18]. During viral replication, APOBEC3 proteins from the previous host are incorporated into budding virions. Once the virus enters a new host cell, it encounters a range of restriction factors acting at different stages of the viral life cycle. These include tetherin, which traps virions at the cell surface [19,20,21]; SERINC3 and SERINC5, which alter viral envelope rigidity [22,23,24,25]; TRIM5α, which causes premature uncoating of the viral genome [26,27,28]; and SAMHD1, which depletes deoxynucleotide pools necessary for reverse transcription [29,30,31]. APOBEC3 proteins, in particular, deaminate deoxycytidine (dC) residues in single-stranded DNA (ssDNA) generated during reverse transcription, converting them into deoxyuridine (dU) [4,13,14,15,16,17,18,32,33,34]. This mutagenesis can result in either complete inactivation of the viral genome or mutations that enable the virus to evade host innate and adaptive immune detection [35,36,37]. Despite these formidable defenses, viruses have evolved specific mechanisms to counteract specific restriction factors [4,19,20,21,22,23,24,25,26,27,28,29,30,31,32,33,34]. Viral Vif, for instance, binds to APOBEC3 proteins in conjunction with host CBF-β, recruiting an E3 ubiquitin ligase complex to polyubiquitinate APOBEC3 proteins, targeting them for degradation before they can incorporate into the virion [35,36,37]. While APOBEC3 proteins are most studied in the context of retrotransposons and retroviruses, they also exhibit antiviral activity against DNA viruses such as herpes simplex virus (HSV), hepatitis B virus (HBV), and parvoviruses [38]. Additionally, there is evidence suggesting that APOBEC3 proteins may restrict certain RNA viruses, including coronaviruses, though their precise mechanisms remain unclear. Collectively, these restriction factors act as barriers to zoonotic transmission, which viruses must overcome to establish infection in new hosts [6,8].

For lentiviruses like HIV and SIV to successfully adapt to a new host species, they must evade the host’s unique restriction barriers, such as APOBEC3 proteins [6]. In primates, the APOBEC3 family underwent remarkable diversification through gene duplication, resulting in seven paralogs (A3A-H) with some containing two fused domains [39,40,41,42,43]. This diversification is thought to have been driven by evolutionary pressures from retroelements and potentially retroviruses. APOBEC3 genes originally evolved from activation-induced cytidine deaminase (AID), an enzyme involved in antibody diversification in jawed vertebrates [44,45,46,47,48]. The initial diversification of APOBEC3 genes occurred approximately 170 million years ago in placental mammals, giving rise to three ancestral subtypes (A3Z1, A3Z2, and A3Z3). Positive selection across taxa has driven further divergence of APOBEC3 genes, often in response to viral pressures, leading to corresponding evolutionary changes in lentiviral Vif [48,49,50,51,52,53,54]. The expansion of APOBEC3 proteins, particularly the A3A-H paralogs [15,40,55,56,57,58], is hypothesized to be a result of this coevolutionary arms race with retrotransposons and retroviruses [5,55,59,60,61,62]. Some APOBEC3 proteins, notably A3A, A3C, and A3H, retain single deaminase domains similar to their ancestral counterparts, while others, such as A3B, A3D, A3F, and A3G, have evolved double domains [34,63,64,65]. In these double-domain APOBEC3 proteins, the N-terminal domain (CD1) is typically inactive but suggested to play a crucial role in substrate binding, three-dimensional diffusion, facilitating ssDNA intersegmental transfer, and binding ssRNA to enter budding virions, while the catalytically active C-terminal domain (CD2) acts through direct mutation. These structural adaptations enhance their antiviral activity, particularly against retroviruses. Among the APOBEC3 family, A3D, A3F, A3G, and A3H exhibit the most potent antiviral effects and are common targets of lentiviral Vif along with A3C [6,32]. Furthermore, A3C, A3D, and A3F are all A3Z2s that maintain a similar Vif-binding site, while A3H has a similar one offset by one α-helix, and A3G-CD1 has a unique binding site that, unlike the others, was recently found to require a nucleic acid intermediate [4,66,67].

The mechanism of APOBEC3-mediated restriction involves binding viral genomic RNA or ssDNA, inhibiting reverse transcription, and introducing mutations into proviral DNA [68]. Each APOBEC3 protein exhibits sequence specificity in its deaminase activity. For example, A3F prefers to mutate *TTC* motifs, while A3G targets *CCC* motifs [69]. The extent and location of these mutations can determine their effects, ranging from complete viral inactivation to the generation of escape mutations that confer drug resistance or immune evasion [70,71]. To counteract these effects, Vif interacts with APOBEC3 proteins to either slow their catalytic rate or promote their degradation prior to viral budding where APOBEC requires binding of ssRNA [5,17,58,72,73]. Importantly, Vif exhibits species specificity, being most effective against APOBEC3 proteins of its native host which underscores the coevolutionary dynamics between host and virus [74,75]. Thus, in addition to a race against both endogenous and zoonotic retroviruses, this adversarial relationship with Vif is believed to have contributed to the rapid evolution and diversification of A3 enzymes in primates [62]. Structurally, APOBEC3 proteins possess a catalytic pocket containing a zinc ion (Zn^2+^) coordinated by a histidine and two cysteine residues with a catalytic glutamate facilitating the deamination reaction. Although the CD1 domain is inactive, it retains the structural components necessary for catalysis and often oligomerizes with other APOBEC3 proteins to enhance DNA editing efficiency through processivity [69,76,77,78,79,80,81,82,83,84,85,86,87,88,89]. Four loops (ℓ1’, ℓ3’, ℓ5’, and ℓ7’) in CD2 form a U-shaped DNA-binding groove with the catalytic pocket positioned at its center with a gatekeeper residue overtop that is able to both occlude or stabilize the entry of DNA in the active CD2 domains [80,82,88,89,90]. Conversely, these loops close around the pocket in A3G-CD1 [84]. Loop 7, in particular, determines sequence specificity through its unique composition [69,76,77,78,79,80,81,82,83,84,85,86,87,88,89]. For instance, A3F contains hydrophobic residues (YYFW), while A3G has hydrophilic residues (YDDQ). The structural arrangement of APOBEC3 domains can vary between globular and dumbbell-shaped configurations, influencing their interaction with nucleic acids and other proteins [91].

The dynamic interplay between host APOBEC3 proteins, viral Vif, and viral nucleic acids highlights the evolutionary arms race that shape host–virus interactions relative to both zoonotic and the following intraspecies transmissions. These coevolutionary pressures have driven the diversification of APOBEC3 proteins in primates and lentiviral adaptations to overcome host defenses. By utilising multifaceted computational approaches to examine the structural and evolutionary variations in A3F, A3G, and Vif across primates and their lentiviruses, we can gain deeper insights into the mechanisms underlying viral zoonosis and host immunity [92]. Although structural computational approaches are only one way of expanding on this diversification, it allows us to observe interactions that have yet to be resolved through more traditional means. Computational approaches, such as those used by King et al., which were solved 2 years prior to the X-ray crystal structure, have proven valuable in predicting protein structures and interactions, offering a framework for studying these complex interactions [93,94,95]. This paper aims to extend our understanding of APOBEC3 proteins and their role in shaping the evolutionary dynamics of host–virus relationships.

## 2. Materials and Methods

### 2.1. Acquisition and Alignment of Sequences

**Figure 1:**
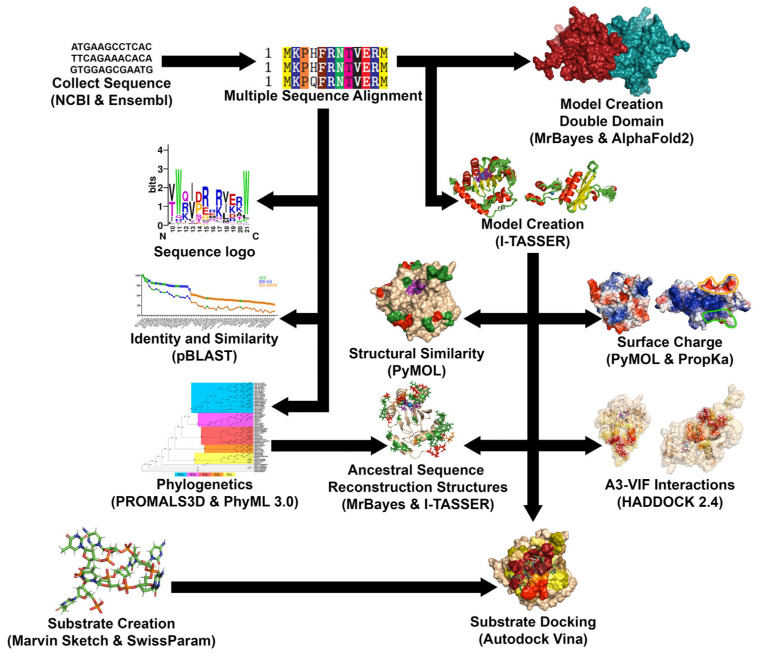
Computational workflow of primate A3FG and HIV/SIV Vif.

We attempted to find all the sequences for all primate A3F and A3G, and all of the sequences of Vif across HIV and SIV (Figure 1). Sequences for all species were attained from NCBI or Ensembl [96,97]. Look at Tables S1–S3 for the individual sources. Alignments were created using Strap bioinformatics suite and WebLogo [98,99,100,101].

### 2.2. Production of Similarity Graphs and Phylogenetic Trees

Graphs of identity and similarity were created utilising NCBI BLASTp under default conditions (as they were sufficient for pair alignment) (Figure 1) [102]. This was done by comparing human genes and HIV-1M NL4-3 genes to all other species and collecting data on the identity, similarity, and gaps in the sequence. This was performed on the full proteins as well as on the domains which had a cutoff for human A3F, which was CD1 M1 to I187 and CD2 L188 to E373, while human A3G was CD1 M1 to I192 and CD2 L193 to N384, and the cutoffs were maintained across other species.

The phylogenetic trees were created utilising the amino acid sequences. Firstly, a multiple sequence alignment was created using PROMALS3D to account for not only the sequence but the 3D structures to which they are related [103]. This was performed under default conditions utilising the sequences of A3F, A3G, or Vif we had found as well as the known protein crystal structures of APOBECs or Vif to aid the construction of the alignment. A3G utilised structures PDB:5K81 [84] and 3E1U [85] for CD1 and CD2, respectively. A3F utilised 2 structures, CD2 structures PDB:3WUS [81] and 5HX5 [104], as no structures exist of A3F-CD1. Vif utilised 2 separate structures, PDB:4N9F [105] and 6P59 [106]. The alignment was then used in the program TranslatorX to create this alignment again in the context of nucleic acids as opposed to the original amino acid alignment (http://translatorx.co.uk, accessed on 8 August 2018) [107]. This alignment was then put through the PhyML SMS suite under default conditions to choose a substitution model to calculate the likelihood of relationship appropriate to each protein [108]. The substitution models chosen were JTT [109], HIVw [110], and GTR [111] for A3F, A3G, and Vif, respectively. Using these amino acid substitution models, the trees were constructed through default conditions of the PhyML 3.0 suite utilising maximum likelihood [112].

### 2.3. Creation of 3D Structural Homology Models

Creation of protein structures of AID/APOBECs through X-ray crystallography and NMR has been generally difficult due to a number of reasons including their genotoxicity, high charged surface area, need for truncations, and mutations for crystallisation. As such, 3D structural homology models were created for each protein utilising Swissmodel to aid in finding proper structural templates as well as create 3D structural homology models to look at the possible pocket conformations (Figure 1) [113,114,115,116,117]. The I-TASSER suite (http://zhanglab.ccmb.med.umich.edu/I-TASSER/, accessed on 8 December 2016) was then used under default conditions to create high quality 3D structural homology models of each species’ A3FG domains as well as Vif across viruses [118,119,120,121,122]. Many templates were utilised for 3D structural homology model creation, with A3F-CD1 3D structural homology models being created from templates 3VOW, 5K81, 5K82, 5K83, and 3VM8 [84,123,124], and A3F-CD2 models from templates PDB:4IOU, 3WUS, 5HX5, 5W2M, and 5ZVB [79,81,82,104,125]. The templates of PDB:5K81, 5K82, and 5K83 [84] were used for A3G-CD1, and 3E1U, 3IR2, 3V4J, 4ROW, and 4ROV [85,86,88,126] for A3G-CD2. To form structures of each A3 catalytic domain, independent domain cut-offs were noted through the context of humans as A3F-CD1 M1 to I187, and A3F-CD2 L188 to E373, in A3G-CD1 M1 to I192 and A3G-CD2 L193 to N384, and the cut-offs were maintained across other species.

Vif 3D structural homology models were created utilising templates PDB:4N9F [105] and 6NIL [127]. Vif 3D structural homology models were taken from M1 to D172 in the context of HIV-1M NL4-3, and this was maintained for 3D location based on the cut-off of current structures at these locations.

Structural templates of all structures were obtained through the protein data bank (http://www.rcsb.org, accessed on 30 September 2016), and all templates and 3D structural homology models were viewed through PyMOL v1.7.6 (http://www.pymol.org/, accessed on 15 September 2016) [128,129]. These models were chosen with Zn in the pocket of A3s and Vif’s HCCH Zn-binding site in HIV/SIV, which were put in place with I-TASSER’s protein–ligand-binding site program COACH [130,131]. These 3D structural homology models were checked for their Cscore (confidence of structure based on threaded template alignments), TM-Score (compared to known structure but insensitive to local error), and RMSD (compared to known structure but sensitive to local error), which were calculated for confidence and similarity with known structures through the I-TASSER Suites (Tables S4–S12). We also constructed Ramachandran plots for each 3D structural homology model to see the ratios of favoured, allowed, and outlier residues where these models maintained greater than 96% favourable results and allowed amino acid angles [132].

A3F and A3G CD2 models were also chosen for having an open catalytic pocket for later DNA docking experiments. Representative species A3s were selected for later dockings so that there would be a human and a representative of each taxon. We then chose the species for having known A3F and A3G sequences and for having known HIV/SIV Vif antagonists, with the exception of the NWMs that have no lentiviral antagonists, so we chose the 2 most diverse species from humans. These representatives were *Pan troglodytes* for GAs and *Papio Anubis* for OWMs, and for A3F *Saimiri boliviensis boliviensis* and A3G, and *Sanguinus labiatus* for NWMs.

The creation of double-domain 3D structural homology models could later be attained through the use of AlphaFold2 after its creation, utilising the monomer_casp14 default conditions with gpu relaxation and all templates up to 2022 on Compute Canada’s Narval server [133,134,135].

### 2.4. Analysis of Structural Surfaces and Charge

Due to variations in the identity and similarity of these proteins and the integral nature of charge in both DNA and inhibitor binding, we set out to determine the charges of these proteins (Figure 1). Analyses of the charges of these proteins were obtained utilising two methods. Firstly, charges of individual protein 3D structural homology models were created using PropKa 3.0 on the PDB2PQR Server (https://server.poissonboltzmann.org/, accessed on 10 September 2018) with CHARMM forcefield at pH7 to ascertain residue protonation states and, from this, calculate the total unfolded and folded charges of the proteins at any pH [136,137,138,139]. Thus, the program was able to determine the protonation state of any individual amino acid based on its interactions with the residues or solvent exposure around it using the charges and atom sizes of the CHARMM forcefield as reference. We tabulated the 4 3D structural homology models (created via I-TASSER) of each domain across all the species at pH 7 to understand the charges. Secondly, PyMOL v1.7.6 vacuum electrostatics were used to visualize the charge by their regions.

### 2.5. Protein-Protein Docking

For an attempt to collate data on the binding sites between Vif, A3F, and A3G, we utilised the HADDOCK2.4 servers under default conditions (Figure 1) [140,141]. This process began through random rotations of the starting structures and rigid body energy minimization, semi-rigid annealing in torsion angle space, and final refinement in Cartesian space with explicit solvent. However, we added further restraints by selecting specific residues of each of the proteins that are known binding sites. For the sites of binding, we utilised those highlighted by Delviks-Frankenberry et al. 2020 for A3G-CD1 and A3F-CD2, and Wang et al. 2018 for the Vif-binding sites [4,41]. These highlighted regions were then chosen again across primates’ A3FGs and HIV/SIV Vif based on their 3D location of amino acids in comparison to the original. Default conditions were the most appropriate for docking as it allowed for an efficient use of resources while also allowing the factoring in of course and fine grain interactions. Furthermore, the parameters allow for a rigid interface to become a semi-flexible interface where the interacting residues (within 5 Å) are more motile while still retaining their core structure to prevent deformation. To aid in this, all hydrogens were removed and then replaced at the end of the process. From an initial 1000 complexes, the ten to fourteen bindings with the lowest energy profiles were chosen, all of which had at least 30 Å^2^ of surface. This is produced through multiple refinements and molecular dynamics steps using OPALs force field to ascertain the positions of the flexible residues, with a final resolution utilising TIP3P water models for final refinements. From this point, PyMOL 1.7.6 was utilised to highlight any amino acid between A3F or A3G and Vif by selecting all amino acids within 4 Å of each other and then these data were collated into charts, graphs, and 3D coloured spectrum analyses of these surfaces.

### 2.6. Creation of DNA Substrates

Docking substrates were created as 7mer nucleotide sequences similar to our previous studies (Figure 1) [142,143]. They were constructed with a 3-base motif in the centre of the sequence as well as repeating nucleotides on either end to give added stability. The substrates created were *taTTCat* for A3F and *ttCCCtt* for A3G. These were chosen since they are well documented as the preferred substrate motifs in humans [69,80,144,145,146,147]. Together, we created both these preferred substrate motifs of A3FG as well as two residues on either side, extending not only the stability of the sequence but also the length of the DNA to better extend down the DNA-binding groove during later docking protocols. The creation of substrates was initiated using ChemDraw Prime v.16.0 (https://perkinelmer-chemdraw-prime.software.informer.com/16.0/, accessed on 19 December 2016) to make the 2D version of the substrate and then Marvin Sketch v.5.11.5 (http://www.chemaxon.com/products/marvin/marvinsketch/, accessed on 10 January, 2017) to assemble it into 3D. From this point, SwissParam (http://swissparam.ch, accessed on 1 November 2018) was used to generate the docking parameters through CHARMM22 force field to account for electrostatic and VDW potentials [148].

### 2.7. Analysis of DNA-Protein Docking Results

After the creation of both the proteins and substrates, four open 3D structural homology models of A3F-CD2, A3G-CD2 for humans and a representative of each primate taxon were chosen (Figure 1). These representatives were *Pan troglodytes* for GAs and *Papio Anubis* for OWMs, and for A3F *Saimiri boliviensis boliviensis* and A3G, *Sanguinus labiatus* for NWMs. These were chosen due to the ability to obtain representatives of the same species (except in NWMs) that, across OWMs and NWMs, were distinct with numerous adaptations compared to humans, while in GAs we wanted a species as close to humans as possible. This research was enabled in part by support provided by sharcnet’s graham cluster of the University of Waterloo (www.sharcnet.ca, accessed on 17 May, 2019) and Compute Canada (www.computecanada.ca, accessed on 17 May 2019). We utilised AutoDock Vina (https://ccsb.scripps.edu/, accessed on 17 May 2019) to ascertain the most energetically favourable conformations of the substrate across the protein’s surface using default parameters (exhaustiveness 8 and energy range 3 kcal/mol) with random seeds utilising its Monte Carlo-based search algorithms [149,150]. This was performed in a 30 Å^3^ box around the α-carbon of the tryptophan of loop 6 in the centre of the substrate groove’s face of the protein, adjacent to the catalytic pocket. This permits the entire substrate groove surface to be interacted with by the DNA docking protocols. For each 3D structural homology model, 11 docking modes were produced per run, and 30 runs were completed in total, leading to 330 docking modes per model. UCSF chimera v.1.11.2 (https://www.cgl.ucsf.edu/chimera, accessed on 15 June 2019) was used to view the conformations of the substrate and its interactions with A3F and A3G [151]. At this point, we tabulated whether the DNA entered the catalytic pocket or not and which nucleobase or portion of backbone entered the catalytic pocket. The data were compiled by grouping the runs in subsets of 6 compiled with all the other models of the species and then averaging across 5 sets per species to gain statistics of which nucleosides fell in the pocket. At this point, all conformations that had the cytidine in the 5th positions within the catalytic pocket were compiled into a list and made into pdb files and placed in PyMOL to ascertain all amino acid residues with which they interacted within 4 Å. This was then compiled for each species based on each amino acid to determine to what extent each secondary structure and residue affected the binding of DNA when cytidine was in the catalytic pocket. This was then able to be used for tabulation and 3D heatmaps.

### 2.8. Ancestral Sequence Reconstruction

For the creation of ancestral sequences, we had already selected our extant species, created a multiple sequence alignment utilising amino acids based on protein structural data, and created a phylogenetic tree (Figure 1). To understand how these proteins evolved, we also required a multiple sequence alignment of the nucleotides and as such used TranslatorX to create the alignment again in the context of nucleic acids (http://translatorx.co.uk, accessed on 8 August 2018) [107]. We utilised MrBayes/2.3.7 employing Markov chain Monte Carlo (MCMC) and used the phylogeny generated prior as a guide tree, as well as the nucleotide alignment, to form our ancestral calculations [152,153,154,155]. The reason why we chose to use this program is because with Bayesian inferences we were able to determine, based on the known relationships of primate species, the probability of any potential extinct organisms’ sequences being a certain residue. We utilised the GTR substitution model where the nucleotide positions of the codons were unlinked, and each run was continued until the standard deviation of split frequencies (estimated marginal posterior probability of a phylogeny splitting into nodes) of <0.01 was realised with a potential scale reduction factor (estimated factor which can reduce the scale of the distribution if simulations were allowed infinite number of iterations) for all parameters of around 1.0. With our phylogenetic guide tree, we chose to calculate dependent on the three nodes from which all great apes, Old World monkeys, and New World monkeys, respectively, descended. This analysis was conducted for each ancestral group with eight nchains (Markov chains) and nruns three times and combined as one phylogeny to create the final result. After the creation of the phylogenies for ancestral sequence reconstruction (ASR), the number of possible sequences with nonsynonymous effects per domain was assessed with the aid of MrBayes Helper (https://seyedjavad.shinyapps.io/MrBayes-Helper/, accessed on 17 March 2022). Three-dimensional structural homology models were created for these possibilities by utilising I-TASSER servers; however, if greater than 20 sequences were available, only the top 20 were subjected to 3D structural homology modelling [118,119,120]. After this, they were placed through the same processes and placed through PropKa [130,131,132,136,137,138,139].

## 3. Results

### 3.1. Evolution of A3F, A3G, and Vif

#### 3.1.1. Vif Contains More Variation than A3FGs in Its Binding Regions

Both A3FGs and Vif have evolved rapidly due to the ongoing host–pathogen race with viruses [6,49,156]. The location of Vif binding on A3FGs varies. While both A3G and A3F have an inactive CD1 and active CD2, Vif binds A3G on the inactive CD1 at the junction of loop 7 (ℓ7) and α4 [4]. In contrast, Vif binds A3F on the active CD2 across the C-term of α2, into ℓ4, and in the middle of α3. Accommodating these diverse binding surfaces, Vif has evolved separate binding surfaces corresponding to A3F and A3G [41]. HIV-1 Vif binds A3F via three disparate regions that come together in 3D, whereas one linear motif constitutes the region that binds A3G. Based on this, we were intrigued to see how these sequences compare across primates and lentiviruses.

**Figure 2:**
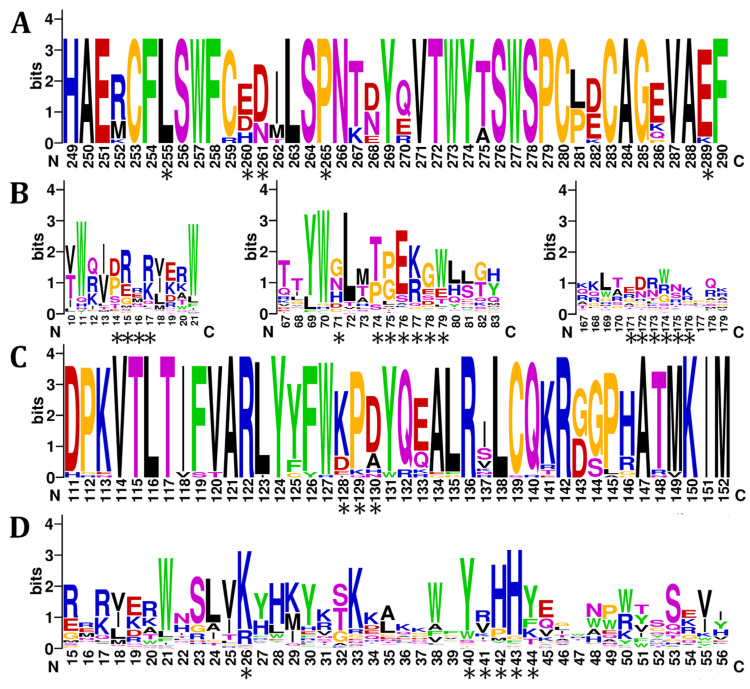
Amino acid sequence logos. All sequences are shown here with heights based on their conservation across alignments of all species across different proteins. Asterisks denote known residues that interact with their targets with either an APOBEC [4] or with Vif [41], respectively. The sequences are A3F denoting Vif-binding sites (**A**), three charts of Vif with A3G-binding sites (**B**), A3G with Vif-binding sites (**C**), and Vif with A3G-binding sites (**D**). Figure created in WebLogo.

Analysing A3F-Vif-binding interactions, we note a host of predicted adaptations amongst both the host and viral proteins. Human A3F-CD2 residues L255, 260DD261, P265, and E289 interact with HIV-1M NL4-3 Vif residues in three binding regions: (1) 14DRMR17, (2) G71, 73TGERDW79, and (3) 171EDRWNK176. Across primates, we notice that positions 255 and 265 are fully conserved, position 289 is highly conserved, while positions 260–261 are variable (Figure 2A). However, when looking to HIV and SIV, we note diversity in A3F-binding regions across residues 14–17, 71, 74–79, and 171–176 (Figure 2B). Of these, the most conserved regions are residues 17 (R/K); 74 (T/P); 75 (G/P); 76 (E); and 77 (R/K). In comparison, very few similarities are seen throughout the third binding region of Vif. Intriguingly, when comparing HIV-1 and HIV-2 lineages, all retain similar motifs across residues 17 and 75–79 corresponding to region 1 and 2, respectively, but not in the third binding region (Figure S1A–C). Thus, when examining the A3F-Vif-binding interactions across species, we note the largest variability localized within Vif, suggesting multiple attempts to circumvent A3s of varying species.

Adaptations can also be seen within the A3G-Vif-binding interactions. Human A3G-CD1 residues from 128–130 (128DPD130) binds HIV-1M NL4-3 Vif residues K26 and 40YRHHY44. Across all primate A3G-CD1, the most common motif is 128KPD130; however, the most common sequence among GA is 128DPD130 (Figure 2C). Across primate Vif, we see high levels of positive residues at residue 26, aromatics at 40 and 44, and mainly histidine at 42 and 43, with other amino acids also seen at lower levels (Figure 2D). When comparing HIV-1 and HIV-2 lineages, we note residues 26, 40, 42, and 43 remain constant, while residues 41 and 44 are quite variable (positive residue/V and Y/K, respectively) (Figure S1D–F). This suggests that there is more consensus in binding of A3G among HIVs than binding of A3F.

#### 3.1.2. Rapid Genetic Evolution of Vif and A3FGs

**Figure 3:**
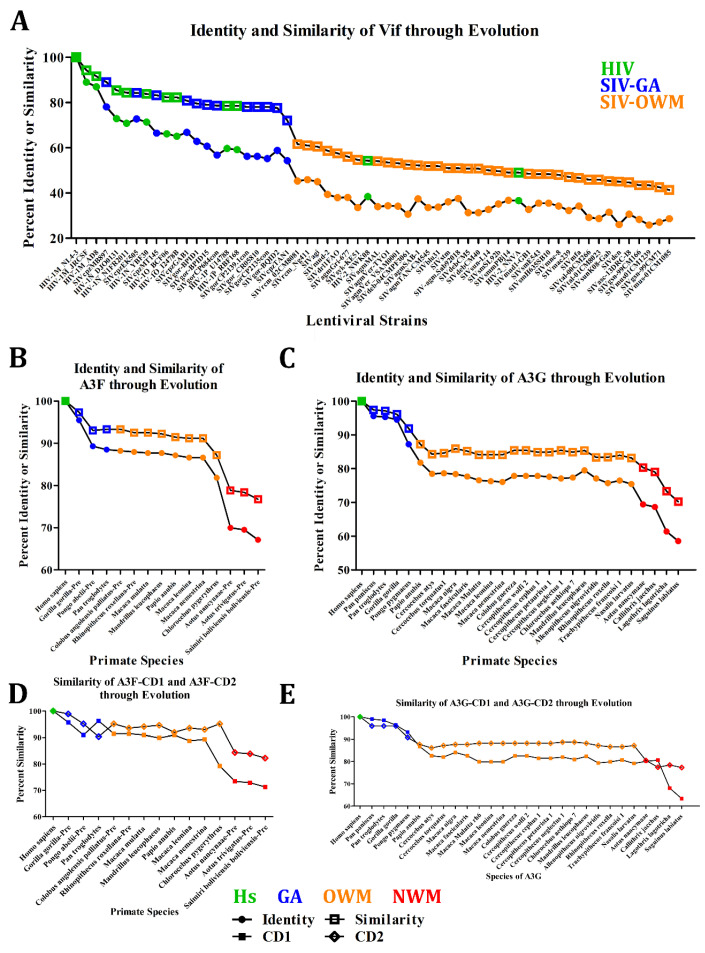
Identity and similarity of Vif and APOBEC proteins. Shown here are the identity and similarities of the amino acid sequences across species of Vif (**A**), A3F (**B**), and A3G (**C**), as well as similarities of A3F (**D**) and A3G (**E**) dependent on domain compared to HIV-1M NL4-3 and *H. sapiens*, respectively. The colour scheme of A3FGs is based on their taxa, while Vif is based on the taxa that is infected, where green denotes humans, blue denotes great apes, orange denotes Old World monkeys, and red denotes New World monkeys.

To explore the evolutionary relationship between Vif and A3FG proteins across species, we analysed their sequence variations. We hypothesized that Vif genes exhibit greater diversity than A3 genes due to their direct targeting by A3s, the error-prone nature of reverse transcription, and selective pressures to overcome host barriers [6,157]. Our analysis revealed that Vif genes vary significantly with similarity to HIV-1M NL4-3 ranging from 94.3% to 73.1% in HIV-1 and SIV of GA, while other lineages, including HIV-2, showed 61.6% to 41.3% similarity (Figure 3A). *Cercocebus atys* Vif genes displayed 54.3% to 48.4% similarity. This variability extends even within Vif genes of viruses infecting the same species. Comparatively, Vif evolution aligns with patterns observed in Env (Figure S2C) and other restriction factor antagonists (Figure S2D–G) but contrasts with the higher conservation seen in Gag and Pol genes (Figure S2A,B). This suggests that while many lentiviral genes, particularly restriction factor antagonists, have undergone significant adaptation, Gag and Pol remain more constrained over 1 million years of evolution [49].

In contrast, A3 genes, particularly A3F and A3G, exhibit less diversity than Vif but still show notable variation across primates. A3F similarity decreases across taxa: 97.3% to 93.3% in GA, 93.3% to 87.2% in Old World monkeys (OWMs), and 78.8% to 76.7% in New World monkeys (NWMs) (Figure 3B). Similarly, A3G exhibits declining similarity: 97.4% to 91.9% in GAs, 87.2% to 83.2% in OWMs, and 80.3% to 79.2% in NWMs (Figure 3C). Comparable levels of divergence are seen in tetherin (Figure S3B) and TRIM5α (Figure S3D). These levels of divergence are greater than those observed in related immune proteins like AID, which only shows a decline from 99.5% to 97.5% across all taxa (Figure S3A). Similarly, there are low divergence proteins like SamHD1 (Figure S3C) and toll-like receptors (Figure S3E–I). This suggests that some restriction factors have diverged more over 33 million years than other immune proteins have over ~400 million years [49,158].

We then wondered if differing evolutionary pressures existed on the catalytic domains (CD1 and CD2) of A3F and A3G. For A3F, CD1 showed greater divergence than CD2 across taxa: similarity declined from 96.3% to 90.9% in GAs, 91.4% to 79.3% in OWMs, and 73.4% to 71.3% in NWMs (Figure 3D). In contrast, A3F-CD2 similarity ranged from 98.9% to 90.3% in GAs, 95.2% to 91.9% in OWMs, and 84.3% to 82.3% in NWMs. Interestingly, in some cases, such as *Pan troglodytes*, CD2 displayed greater divergence than CD1, hinting at species-specific evolutionary pressures. For A3G, CD1 generally showed greater variability, with similarity declining from 98.9% to 93.1% in GAs, 86.8% to 79.2% in OWMs, and 80.6% to 63.4% in NWMs (Figure 3E). Conversely, A3G-CD2 remained more conserved: 95.9% to 90.8% in GAs, 88.7% to 86.2% in OWMs, and 80.5% to 77.3% in NWMs. This divergence in A3G-CD1 is particularly pronounced in species like *Lagothrix lagotricha* and *Saguinus labiatus*, while GAs and *Callithrix jacchus* had greater diversity in CD2. Despite these divergences, the catalytic residues in both A3F and A3G remain 100% conserved across species, underscoring the importance of preserving enzymatic function. The observed differences suggest that non-catalytic CD1 domains, being inactive, are more prone to evolutionary adaptation than CD2, which plays a catalytic role. Notably, A3F-CD2, targeted by Vif, is less affected by evolutionary pressures than CD1. Conversely, in A3G, although CD1 is the primary target of Vif, CD2 exhibits greater diversity in GAs, reflecting selective pressures that extend beyond Vif interaction alone.

When comparing A3F and A3G, their CD1 domains exhibit similar levels of divergence across most taxa, except for highly divergent NWM species. CD2 domains, however, differ between the two proteins, with A3G-CD2 displaying greater divergence than A3F-CD2 in OWM and NWM. This divergence may relate to their substrate specificities: A3G uniquely targets *CCC* motifs, while A3F acts on *TTC* motifs, aligning with other APOBEC3 proteins like A3A, A3B, A3C, A3D, and A3H [69,144,145,159,160,161,162]. Together, these findings highlight how selective pressures shape A3 gene evolution differently across domains and species. The non-catalytic CD1 domains, being less constrained by functional requirements, are more susceptible to mutations and adaptations. However, catalytic CD2 domains also experience specific pressures, particularly in A3G, where unique interactions with viral and host factors drive greater diversification. These patterns suggest that A3 proteins balance the need for functional conservation with the flexibility to adapt to varying evolutionary challenges.

#### 3.1.3. Overall Structure of Vif from HIV Lineages and Primate A3FGs Maintained

**Figure 4:**
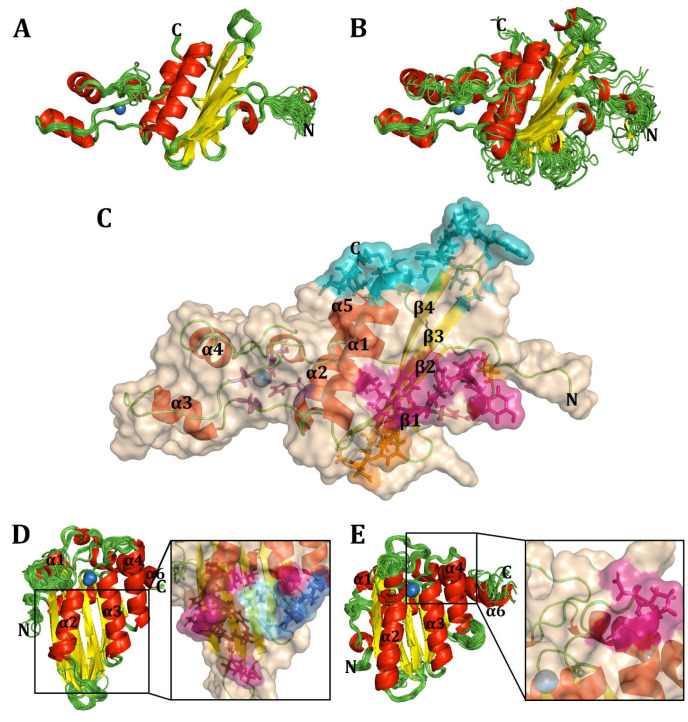
Structures of Vif and A3FGs across species. All structures are shown in cartoon format with colours based on secondary structure with loops (green), α-helices (red), and β-strands (yellow), except for zinc, which is shown as a sphere (sky blue). (**A**,**B**) Vif is shown for great apes as well as Old World monkeys. (**C**) Vif 3D structural homology model of HIV-1M NL4-3 shown as above with surface coloured (wheat) and the HCCH motif interacting with Zn shown as sticks (purple). The binding sites for A3F (cyan), A3G (hot pink), and A3H (orange) are coloured separately as sticks as noted in Wang et al. 2018 [41]. (**D**,**E**) The structures of A3F-CD2 and A3G-CD1, respectively, with their Vif-binding regions highlighted as in Delviks-Frankenberry et al. 2020 [4]. (**D**) The highlighted region of A3F shows the regions known to bind Vif (magenta), regions associated with Vif (brown), regions known to bind CBF-β (slate), and regions associated with CBF-β (cyan). (**E**) The known Vif-binding sites are shown as magenta.

To investigate how sequence variations in Vif and A3FGs affect their structures, we hypothesized that Vif would exhibit structural differences, as studies have suggested variations in binding and Zn requirements [163]. Few Vif structures have been resolved, with existing examples including HIV-1 and SIVrcm, leaving gaps for others such as HIV-2 and many SIV lineages [105,106,127]. Notably, mutational studies suggest that HIV-2, which infected humans via *Cercocebus atys*, may not require Zn binding by the HCCH domain and might interact with A3s differently [164]. Using I-TASSER, we predicted structures of A3FGs and Vif [118,119,120]. For lentiviral Vifs infecting great apes (GAs), their tertiary structures are largely conserved with respect to the known Vif structures (PDB:6NIL and 8E40), with most residues overlapping across viruses (Figure 4A) [66,127]. Variations primarily occur at the N-terminus, which forms a loop wrapping around the β-sheet, and at the α-turn near the HCCH motif, where HIV-2 features a loop instead. In Old World monkey (OWM) Vifs, structural differences are more pronounced, particularly in all looped regions, except ℓ9, and the α-turn and α-helices, except for α2 and α3 (Figure 4B). For instance, α1 frequently disrupts prematurely at its termini with complete breakdown in SIVolc, and in some SIVs, the disruption occurs at the α-turn near Zn transitions into an α-helix. Variability in α4 includes differences in length or extra turns compared to HIV Vifs. Current models lack resolution for the Vif C-terminus, which is also missing from known structures. Examining known binding sites on a 3D structural homology model of HIV-1M NL4-3 (Figure 4C), regions interacting with A3F (cyan), A3G (hot pink), A3H (orange), and Zn in the HCCH domain (purple) show significant variation in SIVs infecting OWMs [41]. However, residues interacting with the E3 ubiquitin ligase complex remain conserved. This suggests that while HIV and its precursors share similarities in A3FG-binding regions, SIV Vifs from OWMs show considerable differences.

We also compared structures of A3F-CD2 and A3G-CD1, hypothesizing these domains would share conserved core structures due to the evolutionary lineage of cytidine and adenosine deaminases [48]. Many APOBEC structures are available, predominantly from humans and macaques, including A3G-CD1 and A3F-CD2 [79,80,81,82,83,84,104,125,127,165,166]. Across primates, A3F-CD2 and A3G-CD1 display remarkably conserved base structures despite evolutionary divergence (Figure 4D,E). A3F-CD2 shows the most variation in loop regions, especially ℓ3’, whereas A3G-CD1’s ℓ3 is shorter, similar to AID structures [94]. Unlike A3F-CD2, A3G-CD1 is inactive, with a smaller catalytic pocket and an extended turn near the catalytic Zn. Additionally, A3G-CD1 features a C-terminal extension of α2, consistent with prior findings [84]. Binding sites noted in Delviks-Frankenberry et al. for Vif on A3F and A3G remain structurally conserved, with minimal structural variation observed at the CBF-β-binding site [4]. These findings indicate that APOBEC regions bound by Vif have retained their core structures while evolving surface modifications.

### 3.2. Structural Evolution of A3F and A3G

#### 3.2.1. The Amino Acid Variation of APOBEC3F and APOBEC3G

**Figure 5:**
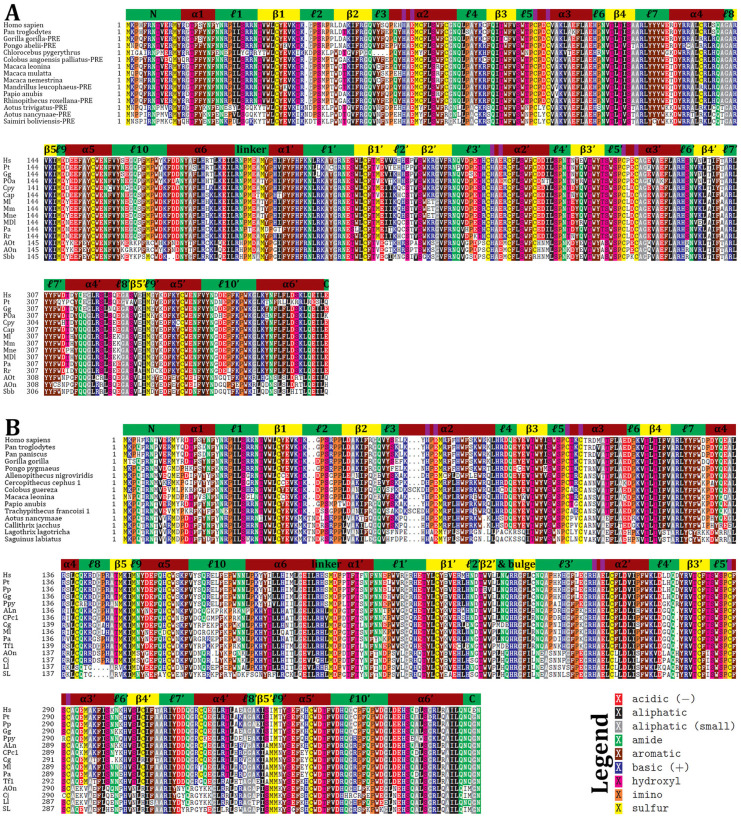
APOBEC3 alignments. Amino acid alignments of A3F (**A**) and A3G (**B**).

The dual-domain APOBEC proteins A3F and A3G contain the inactive pseudocatalytic CD1 domain and the active catalytic CD2 domain. Vif targets A3F-CD2 and A3G-CD1 specifically for degradation [80,81,167]. Alignments of A3F (Figure 5A) and A3G (Figure 5B) show similarities between the two domains, including conserved catalytic residues (HxE and CxxC motifs) in both active and inactive domains. Earlier studies focused on A3F and A3G in humans, African green monkeys, and macaques but revealed evolutionary pressures, such as sequence variation at position 127 in A3G but not in A3F [168]. Variation exists not only across species, but also polymorphisms exist within certain species [49,53].

Primates are categorized into three taxa: great apes (GAs), Old World monkeys (OWMs), and New World monkeys (NWMs). We hypothesized that alignments of A3F and A3G would show evolutionary adaptations while maintaining functionality. Alignments included 15 species for A3F (4 GAs, 8 OWMs, and 3 NWMs) (Figure 5A) and 15 species for A3G (5 GAs, 6 OWMs, and 4 NWMs) (Figure 5B). Additionally, 38 A3G sequences were identified with 23 additional OWM sequences (Figure S4). Adaptations were most pronounced between GAs and NWMs, separated by 33 million years of evolution, yet each species retained unique adaptations within otherwise conserved regions [49,53]. A key feature distinguishing A3F-CD1, A3F-CD2, and A3G-CD1 (A3Z2A subtype) from A3G-CD2 (A3Z1 subtype) is the presence or absence of specific motifs. A WF motif in α-helix 2, five residues after the catalytic glutamate, and an RI/RL motif at the start of ℓ7 characterize these subtypes [169]. Variations include a shift to RF in A3F-CD1 of some OWMs (e.g., macaques and *Rhinopithecus roxellana*) and an RI motif in A3G-CD1 of *Saguinus labiatus*.

Structural elements interacting with ssDNA, particularly loops 1’, 3’, 5’, and 7’, showed varying degrees of conservation and adaptation across taxa [4]. Loop 1 of the CD1’s, from α1 to the gateway residue, contains polar and positive residues with a hydrophobic PIL motif in the middle. In A3F-CD1, loop 1 sequences in NWMs and *Chlorocebus pygerythrus* are distinct. A3F-CD2’s loop 1 is more conserved and positively charged, while A3G-CD2’s loop 1 is highly variable across taxa. Gatekeeper residues also vary. In A3F-CD2 and A3F-CD1, GAs and OWMs typically retain arginine, while NWMs use lysine or glutamine. A3G-CD1 gatekeepers are arginine, except in macaques (leucine), while A3G-CD2 consistently uses histidine. Loop 3, spanning four residues in A3F-CD1 and A3G-CD1, is variable after the initial valine. A3F-CD2’s loop 3, 9 residues long, is conserved except in NWM, whereas A3G-CD2’s 13-residue loop 3 is highly variable but remains polar and positively charged. Loop 5 is typically SWSPC across APOBEC proteins but is SWTPC in A3F-CD1, similar to A3B-CD1 in most primates. Some NWM variants of A3F-CD1 and A3G-CD1 display SWNPC, resembling A3D [166,170]. Loop 7, associated with substrate specificity, contains an RLYYFW planar motif in most A3G-CD1 and both A3F domains. In contrast, A3G-CD2’s loop 7 is RIYDDQ, exhibiting a more polar and negatively charged composition.

#### 3.2.2. Structural Variation of A3F and A3G Across the Species

To understand the impact of sequence diversity on the 3D structures of A3F and A3G, we examined structural variations across species. Prior research established that human APOBEC3 proteins share a similar overall structure, including insights into A3F-CD2’s DNA-binding mechanism, the positioning of its catalytic residues, and the Zn^2+^ cofactor pocket [79,81,82,104]. Furthermore, the negative patch on α-helices 2′, 3′, and 4′, which interacts with HIV-1 Vif, is also well characterized. However, structural data for A3F-CD1 and the Z2a subtype, which includes A3BD-CD1, were absent at the project’s onset [80]. Thus, insights were inferred from related Z2 domains, such as A3C and A3G-CD1. We hypothesized that while Vif-binding regions might exhibit significant variation across taxa, the DNA-binding and oligomerization sites would remain relatively conserved.

**Figure 6:**
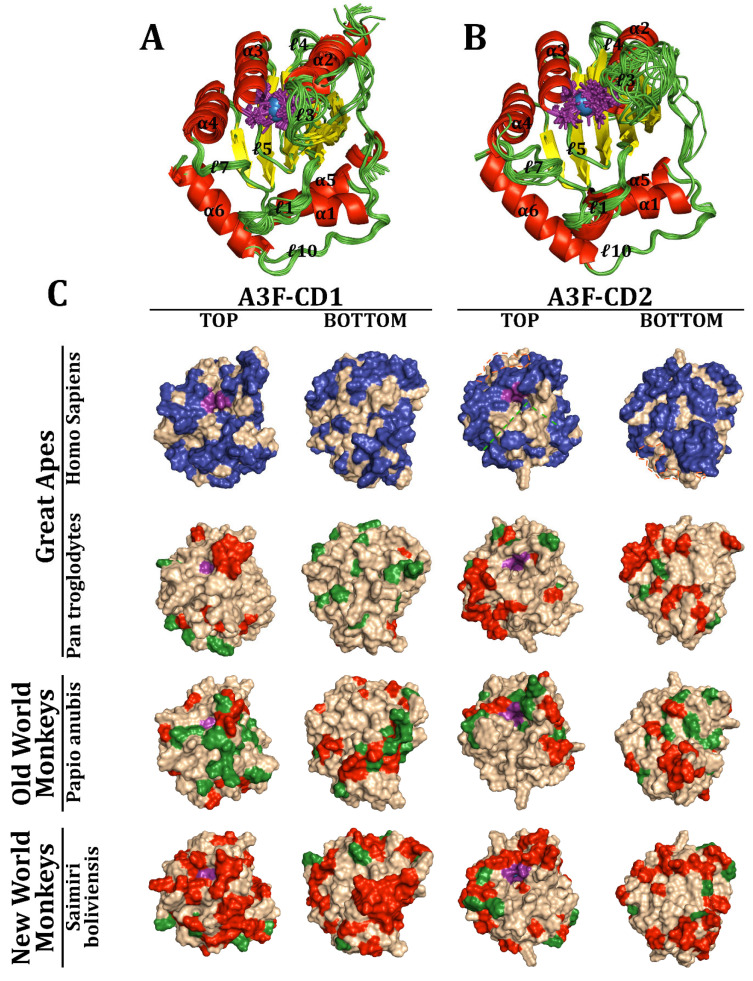
APOBEC3F structure and diversity. Ribbon structures of A3F-CD1 (**A**) and A3F-CD2 (**B**). The surface structures (**C**) of A3F-CD1 and CD2 with representatives of GAs, OWMs, and NWMs are shown from the top (for the DNA-binding groove), and the bottom. The colour scheme shows purple for the catalytic residues, wheat for the unaltered amino acids, and blue for all amino acids in humans that have adaptations across primates while across other primate representatives show adaptations in comparison to humans in red and adaptations compared to other members of the same taxa in green.

Ribbon structures of A3F-CD1 and CD2 were generated and superimposed for comparison across species, including 60 models for each domain (Figure 6A,B). Both domains share six α-helices, five β-strands, and ten loops, along with N-terminal and C-terminal loop extensions on CD1 and CD2, respectively. This aligns exactly with the known experimental models of A3F-CD1 [79,80,81,82,104,125,127]. While α-helices and β-strands showed high structural conservation, loops were more variable. For instance, loop 3 in CD1, which is four residues long with a three-residue extension on α2, differed markedly from CD2, where it extends to nine residues and protrudes into solution without rigid structural support. Despite these variations, key catalytic residues and the Zn^2+^ coordination pocket remained invariant, highlighting their evolutionary stability.

Surface analyses of A3F-CD1 and A3F-CD2 revealed extensive adaptations across the three taxa, including changes around the catalytic pocket and on distal surfaces (Figure 6C). On CD2, variations appeared on the protein’s back, away from the DNA-binding groove. Notably, human A3F-CD2’s DNA-binding region (especially loops 5’ and 7’) was highly conserved, with minimal changes at the C-terminal end of loop 7’. Surprisingly, the human Vif-binding region displayed little variation; only two residues, I262M and C259R, diverged in New World monkeys (NWMs) and *S. boliviensis*, respectively. Most of these adaptations occur on loops and α-helices that exist on the surface of the protein. When focusing on specific taxa, *Pan troglodytes* exhibited localized changes in CD1 near the catalytic pocket (e.g., loop 3) but fewer elsewhere. In CD2, adaptations were more pronounced away from the DNA-binding groove and on β-strands at the back of the protein, though the critical Vif-binding residues (291LAR293, E321) remained largely conserved, with the exception of E321K in *Macaca* and *Mandrillus* lineages. Similarly, Old World monkeys (OWMs) like *Papio anubis* showed adaptations in β-strands and the loop 7’ distal end at D313H but retained stability in the DNA-binding groove. NWMs, such as *S. boliviensis*, displayed widespread structural changes, including within the Vif-binding region (C259R and I262M). Despite these modifications, the core structure of A3F and its DNA-binding regions remained intact across species.

**Figure 7:**
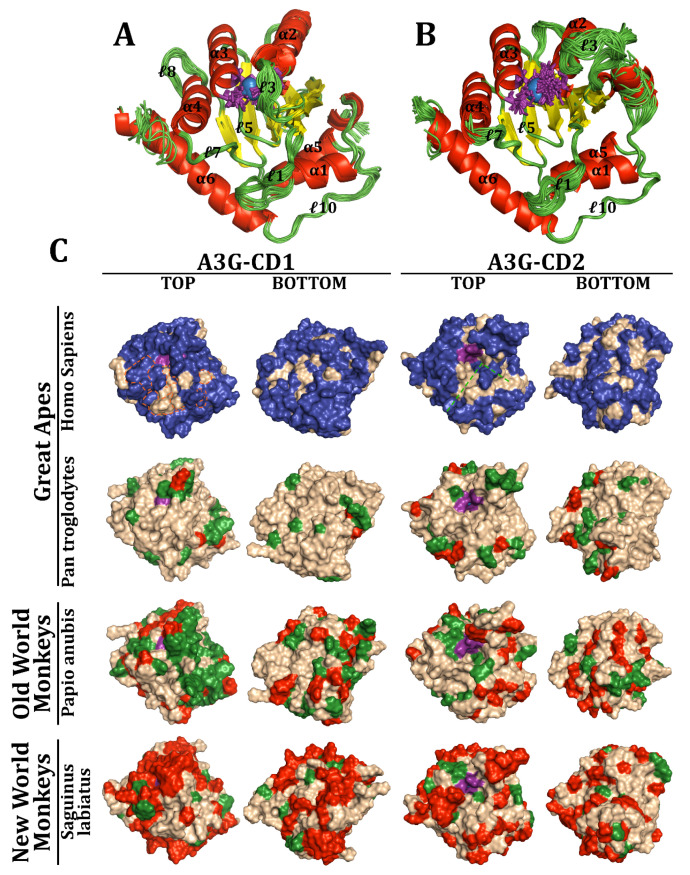
APOBEC3G structure and diversity. Ribbon structures of A3G-CD1 (**A**) and A3G-CD2 (**B**). The surface structures (**C**) of A3G-CD1 and A3G-CD2 with representatives of GAs, OWMs, and NWMs are shown from the top (for the DNA-binding groove), and the bottom. The colour scheme shows purple for the catalytic residues, wheat for the unaltered amino acids, and blue for all amino acids in humans that have adaptations across primates while across other primate representatives show adaptations in comparison to humans in red and adaptations compared to other members of the same taxa in green.

Given the sequence diversity of A3G, we investigated its 3D structure, hypothesizing that Vif-binding regions would exhibit greater variation than DNA-binding regions. Ribbon structures of A3G-CD1 and A3G-CD2, superimposed across 144 and 152 models, respectively, revealed a similar structural framework to A3F (Figure 7A,B). Both domains retained six α-helices, five β-strands, and ten loops. While α-helices and β-sheets were conserved across species, including with respect to the experimental structures, loop 3 in both domains displayed significant backbone variation [84,85,86,165,166,171,172]. In humans, loop 3 in CD1 is 4 residues long, interacting with a three-residue extension of α2, whereas CD2’s loop 3’ extends to 13 residues.

Surface analysis revealed substantial interspecies adaptations in A3G (Figure 7C). In humans, CD1 showed significant changes around the catalytic pocket and the back of the domain, while CD2 exhibited fewer variations near the DNA-binding groove and the back. Loop 7’, critical for substrate specificity, remained mostly conserved except for minor variations at its proximal end away from the groove. In *P. troglodytes* and other GAs, CD1 displayed extensive adaptations, particularly in the Vif-binding region (e.g., Y19D, P129Q), while CD2 showed clustering of changes away from the DNA-binding groove. OWMs, particularly *P. anubis*, showed variations in CD1’s ℓ3 across all residues and Vif-binding regions, including D128K, while maintaining CD2’s DNA-binding region stability. NWMs, such as *S. labiatus*, displayed widespread changes across both domains, particularly in the Vif-binding region (Y59H, F126Y, D128K, and P129K). Although the DNA-binding region of A3G-CD2 remained largely conserved, NWM’s modifications in loop 7’ could influence substrate specificity.

Across both A3F and A3G, the catalytic core and DNA-binding regions are strikingly conserved, reflecting their critical role in maintaining protein functionality. Variations are more pronounced in surface residues, particularly in the Vif-binding regions, which may reflect evolutionary pressures from host–pathogen interactions. In NWMs, extensive adaptations in both A3F and A3G highlight the evolutionary divergence over 33 million years [49,53]. The structural stability of the catalytic pocket and DNA-binding grooves across taxa underscores their functional importance. Meanwhile, surface adaptations, particularly in loops and Vif-binding regions, suggest evolutionary flexibility, enabling species-specific responses to selective pressures. Together, these findings illuminate the evolutionary dynamics of APOBEC3 proteins and their role in antiviral defense across primates.

#### 3.2.3. The Role of Charge Variations in Vif and A3 Proteins

Understanding the charge of proteins, much like their structural characteristics, is critical for grasping how they interact with other proteins and their environment. Protein charge plays a pivotal role in determining the dynamics of these interactions. In this context, we hypothesized that lentiviruses affecting the GA lineage would exhibit similarities in charge profiles, while the A3FGs would show respective charge similarities due to their overarching structural commonalities.

**Figure 8:**
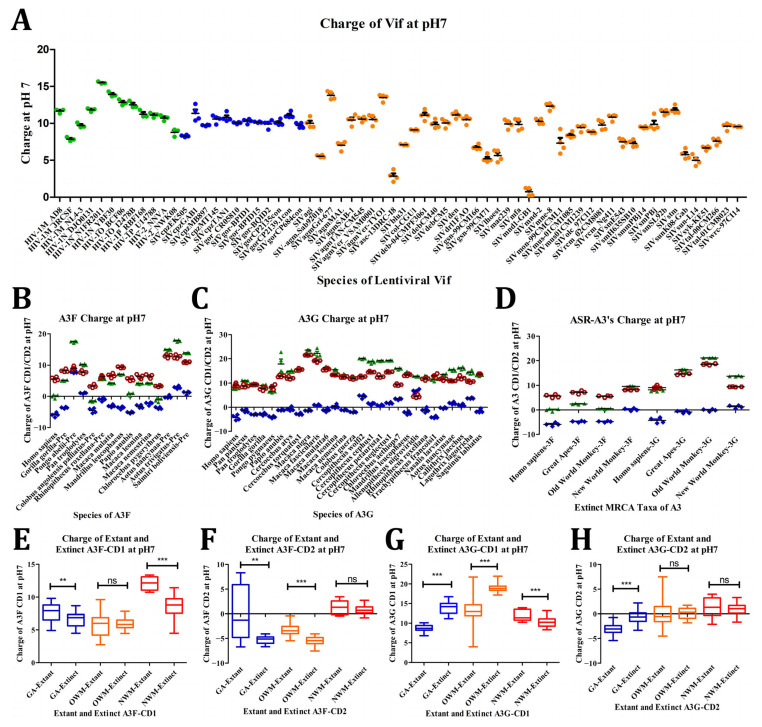
Charge of Vif and APOBEC3FG. The charge of Vif (**A**), A3F (**B**), A3G (**C**), and A3FG ASR models (**D**) at pH7. Vif (**A**) graph is coloured by HIV/SIV host with humans (green), great apes (blue), Old World monkeys (orange), and New World monkeys (red), while A3FG models are coloured by domain with CD1 (red), CD2 (blue), and double domain (green). The charges of extant and extinct ASR models were produced for A3F-CD1 (**E**), A3F-CD2 (**F**), A3G-CD1 (**G**), and A3G-CD2 (**H**), where they are coloured by taxa with great apes (blue), Old World monkeys (orange), and New World monkeys (red). [ns = not significant, *** *p* < 0.0001, (**E**) ** *p* = 0.0019, (**F**) ** *p* = 0.0024].

To investigate, we began with Vif proteins. Our analysis revealed substantial similarities in Vif charge profiles across SIVs infecting GA lineages, whereas there was 2 to 4 times greater variation within HIV and SIVs of OWMs (Figure 8A). Specifically, the charge at pH 7 for SIVs of *Pan troglodytes* and *Gorilla gorilla* ranged from 8.29 to 11.35, for SIVs of *Cercocebus atys* from 7.27 to 11.50, and for HIV in humans from 7.85 to 15.51. In contrast, other SIVs of OWMs displayed a broader range from 0.74 to 13.79. This variability highlights the extensive diversity in SIVs infecting various OWMs with relatively higher similarities among *Cercocebus atys*, *Pan troglodytes*, and *Gorilla gorilla*. When HIV entered human populations, this diversity expanded, suggesting that charge variation was essential for viral survival and replication in humans, even though A3s exhibited limited changes in charge.

Next, we examined the structural impact of these proteins on their charge regions over evolution. Structural analysis of the binding regions in HIV-1M NL4-3 Vif showed a prominent negative region around the A3F-binding site, while HIV-2 NNVA Vif exhibited a smaller negative region, with much of the surrounding area being positive or slightly positive (Figure S5A,B). In HIV-1M NL4-3, the negative charges originated from residues D14, E76, D78, and 171ED172, countered by positive charges from R77, R15, and R17, the latter two interacting directly with E76 and 171ED172, respectively. Conversely, HIV-2 NNVA’s positive charges came from R18 and R174, with a negative charge from D175 and neutral charges from W73 and W81. The A3G-binding regions in both HIV-1M NL4-3 and HIV-2 NNVA Vif remained positively charged due to lysines and histidines. Interestingly, Vif proteins across lentiviruses demonstrated charge variation in their A3FG-binding regions, with fewer changes noted between SIVgor BPID and HIV-1M NL4-3 or HIV-2 NNVA and SIVsm SL92b, reflecting their close phylogenetic relationships (Figure S6).

Shifting focus to the A3 proteins, we examined how surface charge variation influenced the domain charges of these proteins. Based on prior knowledge of human A3 proteins, we hypothesized that the CD1 domains of A3F and A3G would remain positively charged, while CD2 domains would exhibit negative charges. PropKa was employed to measure the charge of 3D structural homology models for CD1, CD2, and dual-domain structures at pH 7, for A3F in Figure 8B and A3G in Figure 8C, respectively. In A3F and A3G, CD1 was consistently more positive than CD2, except in *Allenopithecus nigroviridis* (OWM) of A3G, where CD1 and CD2 had charges of 4.48 and 6.61, respectively. Remarkably, *Pongo abelii* A3F domains showed the smallest charge difference between CD1 and CD2 (1.265), contrasting with average differences of 9.166 for A3F (ranging 2.13–22.86) and 12.68 for A3G (ranging 1.265–13.06). The largest difference occurred in A3G of *Macaca nigra* (22.85). CD1 charges across A3F ranged from 3.25 in *Colobus angolensis* (OWM) to 12.86 in *Aotus nancynaa* (NWM), while CD2 charges ranged from slightly positive to negative, typically between 2.86 in *Aotus trivigatus* and −5.92 in humans. Notably, excepting *Allenopithecus nigroviridis*, CD1 and CD2 charges varied from 21.52 in *Macaca nigra* to 7.65 in *Gorilla gorilla*, and 4.46 in *Cercopithecus wolfi* to −4.29 in humans, respectively. Dual-domain models of A3F often exhibited intermediate charges between CD1 and CD2, except in *Pongo abelii*, *Pan troglodytes*, and the three NWM species. For A3G, dual-domain charges typically aligned with or exceeded CD1 charges, a pattern particularly evident among *Cercopithecus* species. Analysing ancestral sequence reconstructions (ASRs) of the most likely ancestor and extant taxa (Figure 8D, and Tables S13 and S14), we found that A3F domains in GAs and OWMs were similar with NWM domains being more positive. In contrast, A3G domains in GAs and OWMs were more positive than their NWM counterparts. A3F domain charges were variable across GAs and OWMs but less so in NWMs, while A3G charges were consistent within GAs and NWMs but varied significantly in OWMs.

These charge distributions have functional implications. Positive CD1 domains, though non-catalytic, facilitate nucleotide interaction, RNA-directed virion incorporation, and DNA strand traversal through intersegmental transfer [35,36,37,63]. The charged surface of human A3F-CD1 (Figure S7B,F) features three positive regions: (A) the N-terminal loop, (B) loop 1 and the C-terminal of α6 in the DNA-binding groove, and (C) loops 2 and 4 with the back of β-strands 2–5. These patterns are not maintained in NWMs, where region (B) is less positive and loop 7 and α4 become positive along with the negative regions. Human A3G-CD1’s charged surface (Figures S5C and S7D,H) retains a large positive region around the catalytic pocket that extends from the junction of α6 and loop 10, across loop1, the side of β1, and around loop 3 and α-helices 2 and 3 to loops 2 and 8, and other small positive areas around β-strands 4 and 5. A3G-CD1’s three negative regions include (A) across α1 and α5 with ℓ10, (B) ℓ7, ℓ10 and α4, and the C-term of α3. These patterns are conserved across OWMs but differ in NWMs, where positive regions (B) become less positive and loop 7 and α4 gain positive charges. Across OWMs and NWMs, charge locations shifted significantly, with some negative regions in OWMs, like *Macaca nigra*, nearly absent (Figure S7A).

CD2 domains are pivotal for DNA binding and sequence specificity [173]. Human A3F-CD2’s surface (Figures S5D and S7C,G) includes one major positive region and two negative regions. Around the DNA-binding groove were the positive region on ℓ1’, ℓ10’, α1’, α5’, and α6’, with negative charges around ℓ3’, ℓ7’, ℓ10’, α2’, α3’, α4’, and α5’. This distribution is largely consistent across GAs and OWMs, while in NWMs, positive and negative regions are redistributed. Human A3G-CD2 (Figure S7E,I) exhibits five positive and four negative regions, with substantial conservation across taxa. The charged structures around the DNA-binding groove consist of positive charges across ℓ1’, α3’, α4’ and α6’, with negative charges across ℓ1’, ℓ3’, ℓ7’, ℓ10’, α1’, α3’, α4’, α5’, and α6’. The CD2 domain charges across A3F and A3G are divergent, which may be a function of the differences between A3 subtypes and may be in part due to how they interact with their respective substrates (Figure S7A).

Finally, we examined charge trends across ASR and extant taxa using PropKa (Figure 8E–H). In A3F-CD2 domains, charges increased in GAs and OWMs but not in NWMs (Figure 8F). In A3F-CD1, GAs and NWMs showed charge increases (Figure 8E). Conversely, A3G-CD1 charges decreased in GAs and OWMs but increased in NWMs (Figure 8G). A3G-CD2 domains showed no significant charge shifts in OWMs or NWMs, though OWM charges expanded in range while GA charges decreased (Figure 8H). Notably, charge diversification in OWM A3G domains aligns with positive selection and OWM expansion [49]. Overall, our findings reveal that Vif and A3 charge regions have been adapted not only in areas where they interact, but also across the entirety of their surface with exceptions to the A3 DNA-binding regions.

### 3.3. Overall A3 and Vif-Binding Modes Remain Similar Despite Adaptation

**Figure 9:**
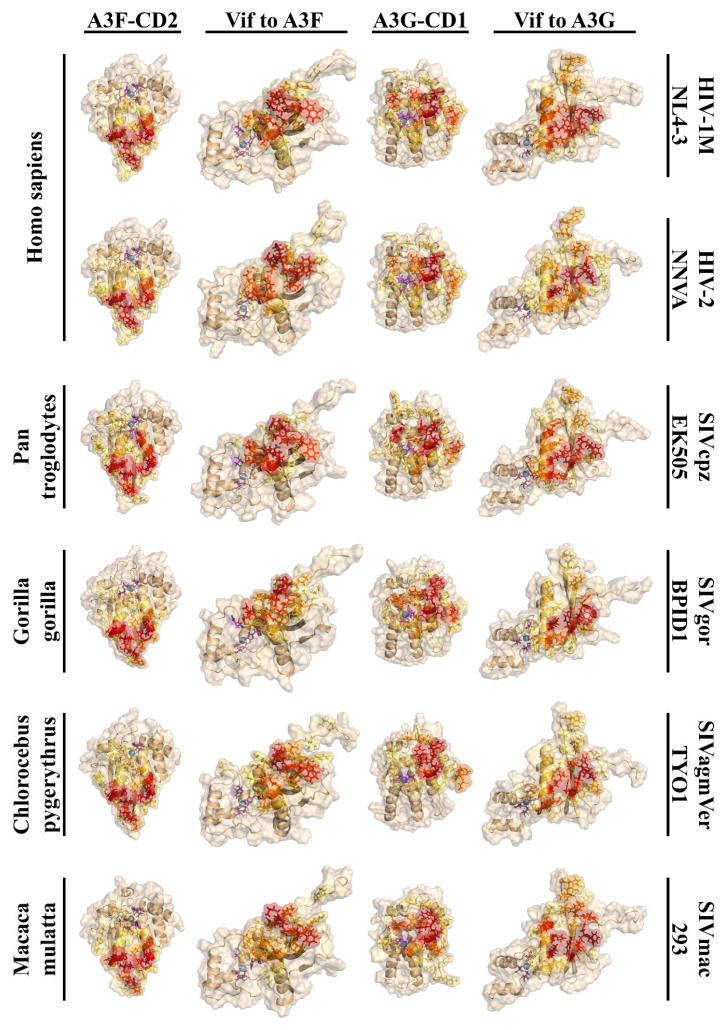
Haddock heatmaps of A3FGs and Vif across primates and viruses. Heatmaps of interactions recorded when docking host A3FG with HIV/SIV Vif. At the top are shown the columns of APOBECs as well as Vif interacting with either A3F or A3G. On the left and right are the primates and the viral strains, respectively. All 3D structural homology models within each column are shown at the same angle for ease of viewing interactions. Cartoons are shown with transparent surfaces, where zinc is shown as a sky-blue sphere; the HCCH motif of Vif and the catalytic residues of A3 are shown as purple sticks; and interactions are shown on a spectrum. The spectrum is shown based on percent interaction with wheat (0%), yellow-orange (25%), orange (50%), red (75%), and firebrick (100%). Sticks of residues were shown under the condition that the residues were interacted with at least 10% of the time.

As we analysed the structural properties of protein cores and their surface charges, our focus shifted to understanding their interactions. Using HADDOCK 2.4, we sought to predict how structural and surface charge variations influenced binding between Vif and APOBEC3 (A3) proteins, specifically A3F and A3G. We hypothesized that these interactions would vary significantly due to differences in surface structure and charge, despite their shared functions in host–virus dynamics. To our surprise, despite sequence variability, secondary structures across these proteins and their modes of interactions remained largely conserved, as indicated by our conformational analyses (Figure 9, and Tables S15–S22). This consistency suggests that the binding interfaces retain functional conservation even amidst evolutionary divergence.

In A3F, interactions with Vif primarily occurred on α2, ℓ4, and α3, with additional binding on ℓ6 (Table S15). On Vif, the primary interaction sites were concentrated on ℓ5, supported by minor interactions on α1, ℓ1, and ℓ11 (Table S16). These interactions were similar across multiple species, but there were notable differences in intensity and frequency, highlighting the evolutionary tuning of these interfaces. Detailed analysis of the A3F-Vif interface revealed host-specific variations (Tables S19 and S20). For A3F-CD2, minor interactions were observed on ℓ2. In GA species, this involved residues 226K/EHH228, whereas interactions in OWM were less pronounced. Significant interactions were concentrated on residues 259CD/ED261 and 263LSPN266 with supporting interactions from S256 and I262. Additional binding was observed on residues 298EF290 and 293RHSN296, while E286 in humans had lesser interactions than K286 in *Pan troglodytes*, showing differential interaction patterns. The Vif-binding interface included three primary regions. The first region, involving residues 14DRMR17 in HIV-1 lineages and 16PGD/R18 in HIV-2 lineages, consistently interacted across species (>50% of the time). These were supported by minor interactions on ℓ1 from residues such as Q12 (HIV-1 lineages) and R/K14 (HIV-2 lineages). The second region, centered on the 76ER/K/Q77 motif in ℓ5, exhibited high interaction frequencies (>70%). Interestingly, we noted a reduction in interactions involving G71 in HIV-1 lineages, whereas the corresponding residue N74 in HIV-2 lineages showed stronger binding. The third major region included residues 171ED172 in HIV-1 lineages and 174RD/N175 in HIV-2 lineages, both maintaining significant interaction levels. During our time on this project, a CryoEM structure of A3F-CD2 and Vif was resolved, for which we calculated the interactions (Figure S8A,B and Tables S19 and S20) [127]. We found that our A3F-CD2 interactions aligned exactly with those of the CryoEM structure, while the Vif portion picked up interactions from H43 and Y44 that were only seen some times in our structures.

For A3G, Vif interactions were concentrated on α4 and ℓ7, with additional binding observed on ℓ1, ℓ3, and α3 (Table S17). While all lentiviruses had high interactions on α1, there was a combination of interactions between β1, α-turn 1, and ℓ3, as well as minor but notable interaction in β3 and ℓ5 (Table S18). Despite some variability, these interactions were broadly conserved across A3Gs and lentiviruses (Tables S21 and S22). Strong interactions were noted on the 127WDPD130 motif, with further strong binding on residues Y125 and Y59 (except in *Chlorocebus pygerythrus*). Additionally, residues 131YQE/Q133 on α4 and 98TK99 and R102 on GAs showed interactions. Loops around the catalytic pocket, particularly ℓ1, displayed heightened interaction, with OWMs showing elevated binding on I26 and S28. The Vif residues for A3G-binding revealed critical interaction points. Known residues such as Y40 and the 42HHYE45 motif in HIV-1 lineages displayed strong binding, with *G. gorilla* uniquely exhibiting interactions on R41. Similar patterns were observed in HIV-2 lineages. Other high-interaction regions included residues K26 and α1 residues N19, 22KR23, and 30YI31, though these varied among lentivirus species. Across all species, residues W70 and 79WH80 consistently interacted with A3G. HIV-2 exhibited additional binding on W51, C54, and Y85, while in SIVagmVer-TYO1, interactions involving W51 and C54 were replaced by M53. During this project, six CryoEM structures of A3G and Vif were resolved, which we analysed (Figure S8, and Tables S21 and S22) [66,67,172]. A3F-Vif structures aligned with our dockings, however, with only one known structure we were unable to analyse with regards to its frequency of interactions. However, between A3G and Vif, there were six structures also containing ssRNA or dsRNA. For the most part, our results including frequency align with theirs save for interactions between A3G ℓ1 and Vif α1 C-term occurring through the RNA.

These interaction patterns highlight a functional necessity for conserved binding topologies, even as specific residues diverge across host species. Despite evolutionary pressure leading to sequence variation, the overall interaction networks between Vif and A3 proteins maintain structural integrity. This balance likely ensures viral adaptation while preserving critical binding capabilities. The results underscore that differences in binding between Vif and A3 proteins are often subtle, manifesting at the level of single amino acids rather than broader structural shifts. This precision reflects the evolutionary co-adaptation of host antiviral defenses and viral countermeasures. While the primary interaction regions are conserved, species-specific modifications optimize binding efficiency, providing insights into the molecular dynamics underlying host–virus interactions. These findings contribute to a broader understanding of how structural and charge variability impacts protein–protein interactions in the context of viral evolution.

### 3.4. A3FG-DNA Interactions

#### 3.4.1. DNA Conformations and the Catalytic Pocket of A3F and A3G

**Figure 10:**
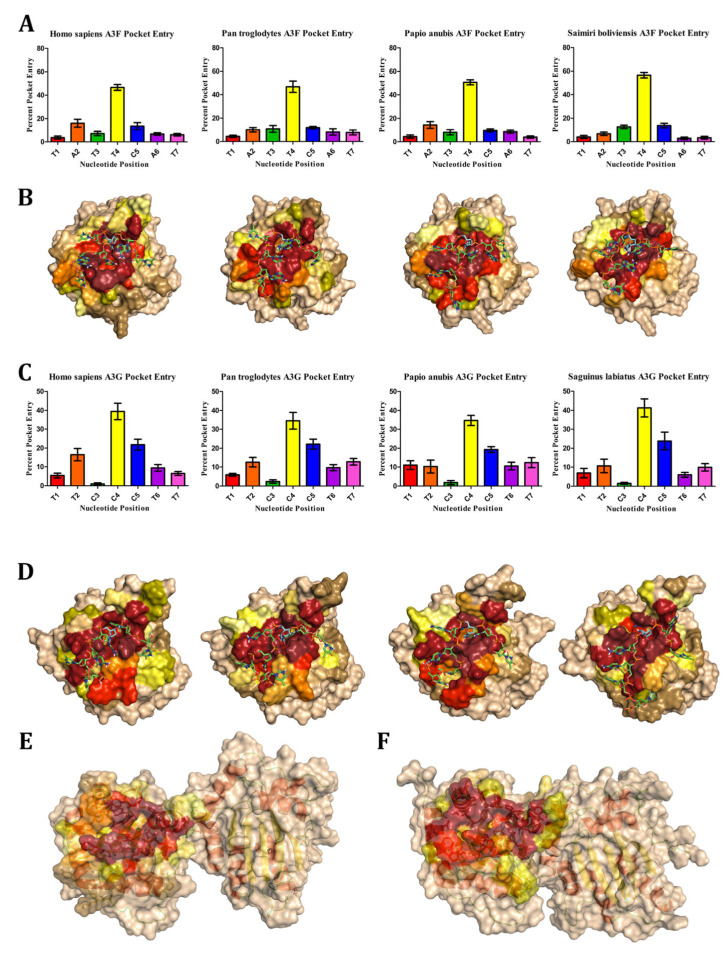
DNA-APOBEC interactions. Analysis of which nucleotide remained in the catalytic pocket most often in A3F species (**A**) and A3G species (**B**). Structural heatmaps of DNA-APOBEC interactions when deoxycytidine is in the catalytic pocket, shown with representatives of the DNA on the surface bound to A3F [left to right: *Homo sapiens*, *Pan troglodytes*, *Papio Anubis*, *Saimiri boliviensis*] (**C**), and A3G [left to right: *Homo sapiens*, *Pan troglodytes*, *Papio Anubis*, *Saguinus labiatus*] (**D**). The heatmap of interaction levels with DNA coloured spectrum from 100% to 0% as ruby, firebrick, red, orange, bright orange, yellow orange, light orange, tv yellow, pale yellow, olive, sand, and wheat. Heatmaps of human double-domain A3F (**E**) and A3G (**F**) are also shown using the heatmap data available from the single domain human heatmaps.

A3F-CD2 and A3G-CD2 display significant diversity in structural homology, surface configuration, and charge across species. However, their core structures and DNA-binding regions remain remarkably conserved. This led us to investigate whether their preferred DNA motifs interact consistently across species. For A3F, we used the motif *taTTCat* and docked it to observe nucleotide interactions with the catalytic pocket (Figure 10A). In A3F-CD2 across *Homo sapiens*, *Pan troglodytes*, *Papio anubis*, and *Saimiri boliviensis*, the thymidine (T4) at position 4 most frequently occupied the catalytic pocket (50.2%), while terminal nucleotides T1 (4.2%), A6 (6.6%), and T7 (5.3%) were the least frequent. After T4, interactions varied: in *H. sapiens*, adenosine A2 (16%), and cytidine C5 (13.4%) were prevalent, while in *S. boliviensis*, T3 (12.6%) and C5 (13.8%) were dominant. *P. troglodytes* showed a mix of A2 (10.0%), T3 (10.9%), and C5 (11.9%), while *P. anubis* favored A2 (14.4%) followed by T3 (8.1%), A6 (8.6%), and C5 (9.7%). Critically, deamination could only occur when a cytidine occupied position 5. Overall, nitrogenous bases entered the pocket in 48.0% of docked conformations, while the backbone (phosphates or riboses) accounted for 6.6%. DNA often remained near the pocket residues (45.2%) and was rarely positioned far from the catalytic pocket (0.2%). For A3G, the motif *ttCCCtt* was used, and docking revealed consistent interactions across *H. sapiens*, *P. troglodytes*, *P. anubis*, and *Saguinus labiatus* (Figure 10C). Cytidine C4 was the most frequently positioned nucleotide in the catalytic pocket (37.7%), followed by C5 (21.8%), which is also the nucleotide most deaminated in vitro. Terminal nucleotides T1 (7.3%), T2 (12.5%), T6 (8.9%), and T7 (10.4%) were consistent, while C3 had the lowest interaction rate (1.7%). The palindromic nature of *ttCCCtt* occasionally reversed the directionality of C3, which was reclassified as C5 for this analysis. Nitrogenous bases entered the catalytic pocket in 57.1% of conformations, with the backbone contributing 12.1%. When nucleotides did not enter the pocket (30.3%), most stayed near catalytic residues, with only 0.5% positioning far away. Despite differences in substrate preferences, A3F and A3G exhibit conserved interaction patterns with their respective DNA motifs across species. This consistency highlights the evolutionary stability of their catalytic mechanisms despite surface-level diversity.

#### 3.4.2. Molecular Interactions of DNA Sequences with APOBEC3F

To understand the interactions between nucleotide chains and the amino acids of A3F-CD2 across species, we analysed dockings where the cytidine at position 5 (C5) of the DNA motif *taTTCat* was within the catalytic pocket. Using PyMOL, we visualized interactions, hypothesizing that despite structural variations, the conserved DNA-binding groove would result in stable interactions across species. Interaction levels were measured as the percentage of contact between DNA and APOBEC proteins across *Homo sapiens*, *Pan troglodytes*, *Papio anubis*, and *Saimiri boliviensis* (Figure 10B). DNA consistently folded along the APOBEC DNA-binding groove, with amino acid interactions visualized in red (high interaction) to wheat (no interaction). Looking first at the secondary structure, most interactions (75.2%) occurred on protein loops: ℓ1’ (29.9%), ℓ3’ (15.3%), ℓ5’ (11.0%), and ℓ7’ (19.0%) (Table S23). Humans showed fewer interactions on ℓ7’ but compensated with increased interactions on ℓ3’. Secondary structures like α1’ (5.4%), α2’ (4.1%), α3’ (5.9%), and α4’ (4.0%) showed modest interactions, while β1’ (1.2%) and β2’ (2.3%) exhibited fewer interactions. Interestingly, α6’ had moderate interactions in great apes (e.g., 2.5% in humans, 3.1% in *P. troglodytes*), which were absent in *P. anubis* and *S. boliviensis*.

On ℓ1’, two key regions consistently interacted across species. In humans, residues 208RK209 and 211YGRNES216 were highly interactive, separated by A210 with moderate interactions (Figure 10B and Table S24). Notable adaptations included *P. troglodytes* substitutions R208L and Y211C. N214, a “gatekeeper” residue, interacted with DNA 100% of the time in humans, *P. anubis*, and *S. boliviensis*. Interactions extended to W217 (or L217 in *P. anubis*) in β1’. ℓ7’, the substrate specificity loop, also exhibited strong interactions. Across species, residues 305RLYYFWD311 (human sequence) showed significant interaction, particularly the hydrophobic region 307YYFW310. *H. sapiens* had less interactions on R305 than all the other species. *P. troglodytes* substitutions from WD to 310QY311 raised site 311 interactions by ~20% compared to other species. Additional interactions occurred at α4’, particularly Y314 (F314 in *S. boliviensis*) and, to a lesser extent, D313 (C313 in *P. troglodytes*). ℓ3’ contained a high number of interactions at N240, V242, and catalytic H249. Moderate to low interaction was seen on Q241, 243DP244, and 246TH247, but interaction was reduced in *S. boliviensis*, which underwent significant adaptations (e.g., human 243DPETH247 to *S. boliviensis* 242GSDPS246). Shifts in interactions were observed at R239 of β2’, which showed high interaction levels in *S. boliviensis* compared to moderate levels in other species. ℓ5’ and α3’ featured conserved residues with moderate interaction levels. Notably, the sequence 277WSPCPEC283 displayed high interaction levels at W277 and catalytic C280. Some species-specific variations included substitutions at 281PE282 to 281LD282 in *P. anubis* and E281 to K282 in *S. boliviensis*. Other secondary structures, like α1’, had consistent moderate interaction levels at I199 (V in *P. troglodytes*, T in *P. anubis*) and 202FH203. In α6’, *H. sapiens* showed a moderate number of interactions with F363, while *P. troglodytes* had additional moderate interaction levels on L364 and R367. *S. boliviensis* and *P. anubis* exhibited only a low number of interactions in 363FL364 and K367.

Despite variations around the edges of the DNA-binding grooves’ amino acid sequences, A3F-DNA interactions are consistent across species. Most interactions occur on specific loops and conserved residues, maintaining the functional integrity of the catalytic pocket and substrate specificity. These findings underscore the evolutionary stability of A3F’s interactions with DNA, highlighting the conserved mechanisms underpinning APOBEC activity.

#### 3.4.3. Molecular Interactions of DNA Sequences with APOBEC3G

To further explore the interactions between nucleotide chains and A3G-CD2 across taxa, we examined its preferred motif (*ttCCCtt*) when the cytidine at position 5 (C5) was within the catalytic pocket. Using PyMOL, we visualized interactions and hypothesized that the conserved DNA-binding groove would result in many consistent interactions across species despite sequence diversity. As shown in Figure 10D, DNA folded across the APOBEC DNA-binding groove in 3D structural homology models with amino acids colour coded by interaction frequency (red indicating the most interactions and wheat indicating none). Analysis of secondary structures revealed that 77.8% of interactions occurred on the protein loops, predominantly ℓ1’ (27.4%), ℓ3’ (20.3%), ℓ5’ (11.4%), and ℓ7’ (18.6%) (Table S25). Loops ℓ1’, ℓ3’, and ℓ7’ displayed consistent interactions across *Homo sapiens*, *Pan troglodytes*, *Papio anubis*, and *Saimiri labiatus*. However, ℓ5’ exhibited slightly reduced interactions in *S. labiatus*. Secondary structures such as α1’ (4.5%), α2’ (4.7%), α3’ (3.3%), and α6’ (4.4%) contributed modestly, while β1’ (1.5%), β2’ (1.3%), and α4’ (2.0%) had lower interaction frequencies. Sporadic interactions occurred across other regions.

In ℓ1’, two regions, 210PWVRG214 and 216HET217, showed high interaction frequencies, separated by a moderate number of interactions at R215 (Figure 10D and Table S26). The first region included adaptations such as L210 in *P. troglodytes*, S213 in *P. anubis*, and both S211 and L213 in *S. labiatus*. In the second region, there were only interactions at Q217 in *S. labiatus*. High interaction frequencies were observed universally at H216, a key “gatekeeper” residue above the catalytic pocket. This was consistent across species, continuing through the next two residues until β1’, where Y219 exhibited moderate interaction frequencies. On ℓ3’, two highly interactive segments were identified: 244NQAP247 and 254EGRH256, including catalytic H257. Residues N244, 246AP247, and H257 consistently showed high interaction frequencies. Notably, *P. anubis* displayed strong interactions at Q245, while *S. labiatus* showed high interaction levels at its S248 adaptation. Moderate interaction frequencies were seen at Q245 (E242 in *S. labiatus*) and 254EG255 (K254 in *P. anubis*). Residues such as 248HK249 (248DI249 in *P. anubis* and 245NN246 in *S. labiatus*) and R256 exhibited lower interaction frequencies. On β2’, position 243 showed moderate interaction levels across species, varying between cysteine (great apes), leucine (New World monkeys), and arginine (*P. anubis*), where interactions were strongest. Catalytic glutamate (E259) consistently maintained high interaction frequencies, while A258 had moderate interaction levels. ℓ7’, the substrate specificity loop, featured high interaction frequencies across the critical motif 315YDDQ318 (*H. sapiens*, *P. troglodytes*, *P. anubis*) or 312YDYR315 (*S. labiatus*). Neighbouring residues I314 and R313 displayed high to moderate and low interaction levels, respectively, influenced by their positioning within the ℓ1’/ℓ7’ interface. Moderate interaction levels extended to α4’, particularly R320 (Y318 in *S. labiatus*). In ℓ5’, the motif 285WSPC288 showed predominantly high or moderate interaction levels across species, though the number of interactions with S283 in *S. labiatus* was low. These interactions extended to α3’, where C291 maintained moderate interaction levels, while residues 289FS290 exhibited lower interaction frequencies. In β3’, low interaction levels were observed at T283 (*H. sapiens*; I280 in *S. labiatus*). Toward the C-terminal region, α6’ consistently displayed high to moderate interaction frequencies at R374 and moderate interaction frequencies at L371, with other interactions occurring sporadically.

The interaction analysis across secondary structures and amino acid residues revealed a highly conserved A3G-DNA-binding interface across taxa. Despite sequence diversity and structural adaptations, the DNA-binding groove consistently mediated interactions, maintaining functional integrity and catalytic activity. These findings underscore the evolutionary conservation of A3G’s DNA-binding mechanism, highlighting its critical role in APOBEC function.

#### 3.4.4. Comparison of A3F and A3G Double Domains Across Ancestral Proteins

One issue with understanding how DNA binds double-domain APOBEC proteins is that the double-domain proteins can exist in globular states, dumbbell states, and transitional states (between the two), which affects how these proteins are able to traverse the genome [63,65,91,165]. This means that DNA can interact with these domains differently dependent on which form is present at the time. As such, we created double-domain 3D structural homology models of these proteins in AlphaFold2, across which the A3Fs (Figure 10E) tended to maintain a dumbbell and the A3Gs (Figure 10F) maintained globular conformations; yet, in both cases, these 3D structural homology models were formed with a similar rotation in orientation with respect to CD2. This rotation is more similar to the recent experimental A3G structures than to the original ones produced in 2020 by Maiti et al. and Yang et al. [65,165,166,172]. Although these 3D structural homology models are static, these conformations would be in constant flux. The conformations of both grooves of A3F and A3G of humans, coloured by their interactions with DNA, show that in these conformations, the nearest place for the DNA to interact would be across the β-sheet of the CD1 domain, which has previously been shown to be a putative RNA-binding region in AID [174]. These are typified by positive charges sandwiched between the positive loops 2’, 4’, 6’, and 8’ as well as the negative charges across the α5’, α6’, and loop 10’ with positive or polar charges across the β-sheet (Figure S7F,H). Together, this shows an alternate path that ssDNA could take along the β-sheets of A3FG-CD1 domains across all species that in AID is known to bind RNA. This β-sheet region is similar across all ancestral A3F-CD1s and A3G-CD1 of GAs, while ancestral A3G-CD1s of OWMs and NWMs have modifications to this region (Figures S9 and S10).

## 4. Discussion

As seen in SIV/HIV, zoonosis requires adaptation to host zoonotic barriers, thereby forming viral variants and later new viral species [6,8]. Such pressures on viruses and hosts can be seen most starkly in the interactions between host barriers and viral antagonists. Throughout this study, we have attempted to understand the coevolution of hosts and viruses through the computational analysis of the evolution of Vif, A3F, and A3G, as well as their interactions with their targets through antagonism and DNA, respectively. Most structures of APOBECs and Vif from prior studies have been limited to humans and several other species, such as *macaca mulatta*, without a comprehensive look across all primate and viral species [67,104,127,165,166]. Furthermore, these studies predominantly showed A3F, A3G, and Vif with mutations or truncations including only one A3 domain present at a time, and only recently have double-domain A3G crystal structures been created. As such, it was difficult to understand the qualities and ramifications of how interactions between these macromolecules across hosts and viruses in a single species, let alone across the evolution of all known primates and their known lentiviral antagonists, occurred. To this end, we noted the high level of genetic diversity across species, including at the A3-Vif-binding sites especially in Vif (Figure 2, Figure 3 and Figure 5), and determined that there are adaptations across the surface of A3s but not in the DNA-binding groove (Figure 4, Figure 6 and Figure 7); that charges have changed across evolution with respect to known Vif-bound domains of A3s, as well as the diversity of charges that exist across Old World monkeys (Figure 8); and the similarities in binding interactions of A3s with Vif and DNA across species given this level of diversity (Figure 9 and Figure 10). Throughout this study, we expanded on these key structural inferences to the coevolutionary dynamic of the A3-Vif host–viral system.

Viruses, with their short life cycles and error-prone reverse transcription, evolve rapidly under host selective pressures [49,157]. Host zoonotic barriers, such as APOBEC3s (A3s), SamHD1, tetherin, and SERINCs, exert strong evolutionary pressure, driving viral adaptations [6]. For example, the stability of host A3s compared to viral Vif binding sites (Figure 2) illustrates this dynamic. Notably, A3G-Vif-binding regions are more conserved than A3F-Vif-binding regions, despite A3G-CD1 being non-catalytic (Figure 2 and Figure S1). All Vif-bound A3 domains, except A3H (A3Z3 family), belong to the A3Z2 domain type (e.g., A3B-CD1, A3C, A3F-CD2, A3G-CD1) [4,41,175]. While human A3CDF proteins share conserved binding sites with Vif, A3G’s unique binding site likely requires fewer viral adaptations to inhibit its function. As Zennou et al. (2006) demonstrated, different species exhibit variable A3 mutational rates and Vif inhibition capacities, reflecting species-specific selective pressures [168]. This may lead to diverse Vif adaptations targeting distinct A3 family members, depending on polymorphisms and their effectiveness against viral defenses [53,176]. Primate A3FG and HIV/SIV phylogenies used prior to creating ASR (Figures S11 and S12) highlight the extensive diversification of both host and viral proteins, which is consistent with findings from previous studies on divergence patterns [5,49,52,176,177]. Reduced gene identity and similarity in A3 sequences (Figure 3 and Figures S2 and S3) further validate these observations. As Sawyer et al. posited, A3G undergoes Darwinian positive selection at viral binding sites, a phenomenon also observed in A3F, tetherin, TRIM5α, and SamHD1, indicating that restriction factors adapt to diverse viral pressures [49].

This study focused on A3F and A3G restriction factors, examining their structural features, surface charge distributions, and interactions with preferred DNA motifs. Amino acid alignments highlight regions of perfect conservation alongside numerous non-synonymous adaptations, especially within catalytic loops, potentially affecting DNA interactions (Figure 5). Interestingly, some sites universally conserved across human A3s [87], such as A3F positions R30, I117, F159, F167, P169, M195, and I301, as well as A3G positions R30, I118, L135, F172, P174, W175, R215, F262, L271, V305, I309, A312, and I337, exhibit variability across taxa. This suggests diverse evolutionary pressures shaping these proteins, leading to conservation across humans that was lacking across some taxa or even in some cases species, although it cannot be determined what pressures caused all of these adaptations.

The amino acid diversity of A3F and A3G spans a broad range (97.3–76.7% and 97.4–70.2%, respectively), contrasting with the highly conserved immune gene AID (99.5–97.5%), which is essential for immunoglobulin DNA somatic hypermutation and class switch recombination, having been conserved back to lampreys (Figure 2B,C and Figure S3A) [158,178,179,180,181]. A3F and A3G, however, function on the front lines of immune defense, targeting lentiviral Vif (both directly and indirectly), and possibly extinct viral antagonists [62]. Evolutionary pressure is more pronounced on CD1 domains, which lack direct catalytic activity, than on CD2 domains, which must retain DNA-binding grooves and catalytic pockets for activity (Figure 3D,E). This dichotomy aligns CD1s more closely with TRIM5α in diversity and CD2s with SamHD1 and tetherin for A3F and A3G, respectively. CD1’s role in RNA binding for HIV/SIV virion packaging and intersegmental DNA transfer likely contributes to its higher diversity compared to the catalytically active CD2 [35,61,78,182]. Notably, lentiviruses do not infect all primates, such as New World monkeys, suggesting species-specific pressures beyond just lentiviral ones [5]. Other retroelements, such as Line-1, are also targeted by A3 proteins, with A3F showing greater effects in humans than A3G [61]. These extant and possibly extinct pressures likely drive the observed protein diversity across taxa. The distinct functional capacities of A3F and A3G, as shown in their species-dependent ability to reduce infectivity, further underline their evolutionary specialization [168]. Overall, CD1 diversity supports its broader functional requirements, while CD2 conservation reflects its critical catalytic role in maintaining sequence specificity and DNA-binding stability.

While innate TLRs and AID, which facilitate antibody creation, show that not all parts of the immune system are similarly pressured, the fact that SamHD1 (which depletes dNTPs) has less positive selection than other restriction factors shown here may denote that presence of antagonists is not the sole determinant of evolution (Figure S3) [29,30,31]. Instead, the positive selection may be due to both immuno-evasion and methods of action, which may place it in direct contact with the viral proteins or genome, as seen in the other restriction factors [20,26]. On the other hand, it is unsurprising that lentiviruses would have high diversity, especially as HIV-1 is noted for its high mutation rate [157]. Nonetheless, noticeably in HIV-1, higher mutation rates have been seen occurring in regions of Gag, Pol, and Env in comparison to restriction factors, which could be due to differences between a zoonotic virus attempting to adapt to a new host relative to an endemic virus resisting pressures in a current host (Figure S2). Fortunately, we were able to obtain a large repertoire of HIV/SIV and primate proteins to compare for these experiments. However, this has led to a limitation where there is high variance in the C-term of Vif alignments, as well as some proteins where we were not able to have sufficient alignment to determine percent identity and similarity. Notwithstanding, our results indicate selective pressures across restriction factors and their antagonists, both past and current, that have led to greater diversities than are commonly seen, even in comparison to other immune genes.

While linear sequences provide valuable insights, protein structure is essential for understanding host–virus interactions. Structural studies of A3FGs and Vif, though limited across species, have shown remarkable conservation in the core structure of these proteins, as evidenced by Figure 3B,C [84,127]. This aligns with findings that all AID/APOBEC family members exhibit a high degree of structural similarity, further supported by ASR 3D structural homology models (Figure 10E,F) [183,184]. The cytidine deaminase superfamily, which includes AID/APOBEC proteins, maintains a conserved core structure across various deaminases, such as guanine, guanosine, and adenosine deaminases, as well as dimeric and trimeric cytidine deaminases [46,48,185,186,187,188,189,190,191,192,193]. Within APOBECs, A3s are classified into Z1, Z2, and Z3 domains, highlighting structural similarities across lineages [80]. For example, A3F and A3G are primarily composed of Z2 domains, except for A3G-CD2, which belongs to Z1. Z2 domains can be further categorized into Z2a (A3F-CD1), Z2b (A3F-CD2), and Z2c (A3G-CD1). Structural comparisons reveal that the pseudocatalytic domains of A3F and A3G (Figure 6A and Figure 7A) retain the APOBEC fold observed in active domains (Figure 6B and Figure 7B). However, modifications in the loops and surface of CD1 disrupt the DNA-binding groove characteristic of catalytically active APOBECs [80,85,88,93,94,95,194,195]. These observations reinforce the evolutionary structural conservation of A3 proteins while highlighting domain-specific adaptations that may influence their functional roles in host–virus dynamics.

Unfortunately, in the case of Vif, there are very few known structures and none of HIV-2; as such, it is more surprising to us that HIV-2 is able to maintain this overall structure given it only retained around 50% sequence similarity [105,127]. Smith et al. 2014 showed that Vif of HIV-2 was able to bind CUL5, even when Zn was not present, and bind to a separate region of A3F and A3G [164]. As for the structures of many SIV-Vifs that infect OWMs, there is very little literature on their structure, save for the structure of SIVrcm-Vif, which has many similarities in overall structure with HIV-1 Vif in the regions that have been determined [106]. As Binning et al. stated, the variations in these unstructured looped regions could have effects on their targeting and would be important, along with a maintained protein fold; however, the disruption of this structure in SIVolc means there is a possibility of a highly variable Vif in this species.

The strength of our 3D structural homology modelling system is that it can accurately predict the structures of these proteins even with low percent identity; however, these are limited by the number of structures that have been previously found. This accuracy has been previously shown in our laboratory when we were able to accurately predict the structure of AID, which was later confirmed by X-ray crystallography [95,146]. As such, it limits our knowledge of the C-term of the protein which has not yet been fully elucidated. When we observe the charges of these proteins in Figure 8A–D and Figures S5–S7, we see that both Vif and A3s have varied in their charges, but it is important to note that this is only considering the charges of the Vif-sensitive domains of A3FGs and all the regions that we can model using the 3D structural homology of Vif. Knowing that SIVs of OWMs have existed longer than those in GAs, as well as the known divergence between OWMs, it makes sense that charge ranges in these Vifs are highly divergent; however, while SIV of *Pan troglodytes* and *Gorilla gorilla* come from the same source, their low charge diversity also is understandable [6]. Intriguingly, the range in charges for HIVs may be due to the multiple lineages that were formed by four separate zoonotic events in the HIV-1s, while HIV-2 comes from nine events from sooty mangabeys. Furthermore, variance in the charges may indicate not only the necessity of adapting to host A3s but also other host mechanisms of the ubiquitination system. Conversely, A3FGs have ranges in their charges across all taxa that could be a result of selection from multiple viruses over evolutionary time, just as today they are known to interact with retroviruses, retrotransposons, HSV, HBV, parvovirus, coronaviruses, and endogenous retroviruses [38,62]. This may explain the trends seen in Figure 8E–H across A3FGs, comparing their extant and ASR charges as different pressures occurred prior to what we know of as the evolution of HIV/SIV [49]. Our phylogenetic analysis employed multiple Bayesian runs rather than relying solely on maximum likelihood or maximum parsimony, enhancing the accuracy of ancestral sequence reconstruction (ASR) and 3D structural homology models. However, due to the extreme diversity of HIV/SIV Vif, we were unable to confidently derive ASR sequences for 3D modelling.

In analysing A3-Vif interactions (Figure 9), we observed that binding regions aligned with the established literature, though amino acid-level variations influenced the nature of these interactions [4,41,175]. This suggests that Vif’s ability to bind A3s across species requires fine-tuned modifications, particularly at non-interacting proximal sites, to maintain functionality. However, it is of note that a limitation of the method we utilised is that completely blind docking is not possible. Regions of interactions must be chosen that could affect our results, which is why we reasoned that a larger aggregate approach would give a clearer picture of the data. Lentiviruses rely on these interactions to navigate zoonotic and interspecies barriers, including polymorphisms [53,156]. Our findings corroborate previous research that Vif interacts with A3F primarily through the α2-3 cleft, though our data highlight a crucial role for CBF-β in stabilizing these interactions [127]. Without CBF-β, Vif compensates by stabilizing alternative binding sites. Interestingly, HIV/SIV-Vif can effectively bind A3G independently of CBF-β, maintaining stable interactions at known surfaces, which suggests reduced reliance on CBF-β for A3G binding [167]. In contrast, HIV-2 Vif targets distinct regions of A3F and A3G, although our results suggest these interactions may converge on similar binding locations [164]. For A3F, binding to modified regions on the first site may initially occur, but subsequent CBF-β binding could destabilize the interaction, preventing degradation. Conversely, HIV-2 Vif-A3G binding appears viable, albeit with diminished strength due to reduced interactions at Vif residues Y41 and H45. Interestingly, Vif also targets PPP2R5 to induce G2/M cell cycle arrest [196]. While this interaction spans a larger region of Vif than A3 binding, it shares four residues with A3F’s binding site and three with A3G’s. Although CBF-β binds Vif in this context, it does not directly interact with PPP2R5—similar to A3G binding but without requiring a nucleic acid intermediate. Notably, these shared residues are more evolutionarily conserved than their surroundings, suggesting that PPP2R5 binding may constrain Vif’s adaptability in interacting with A3F and A3G.

Detailed structural observations further elucidate Vif-binding specificity. For A3F, Vif targets CD2 on two negatively charged and hydrophobic patches predominantly located on α2’, α3’, and α4’ helices [79,80,81,82,104,197]. These binding sites are comparable to those seen in A3C and A3H [80,81,123,198,199]. In A3G, Vif binds near what would have been the catalytic groove if the domain were active, unlike other APOBECs [156,167,198]. Across species, A3G exhibits significantly more adaptations in its Vif-binding regions than A3F, reflecting a broader divergence in A3G-binding sites (Figure 6C and Figure 7C). While A3F-binding regions also show occasional adaptations, these occur less frequently compared to A3G [51,52,156,167,177,200]. This diversity highlights the evolutionary arms race between host A3 proteins and viral Vif, where both must adapt to sustain their respective functions. For A3F, this involves precise interactions with Vif in the α2-3 cleft and stabilization through CBF-β, while A3G demonstrates a more autonomous binding capability with Vif, particularly in its catalytic groove-adjacent region. The variability in Vif-binding adaptations across species emphasizes the dynamic interplay of these proteins under selective pressures, ensuring the persistence of lentiviral survival strategies amidst diverse host defenses.

Our understanding of binding is currently hampered by lack of clear 3D structural homology models of the full-length Vif since all 3D structural homology models are missing the C-term in which particular parts of both Vif-binding regions are contained (Figure S8 and Tables S21 and S22) [175]. Furthermore, the lack of CBF-β across multiple species in A3F leads to a lack of information on other host–virus interactions seen in humans through 6NIL [127]. However, given the differences between our human 3D computational models and those of the CryoEM structure, it is very likely that these interfaces remain similar across all species. It was also discovered recently that Vif-A3G binding requires ssRNA for proper binding, which is also integral to the method used for A3G to be transported to the budding virion [65,66,67,172]. This could mean that some of the residues we noted on A3G ℓ1 and Vif α1 are not as interacted with as we thought and instead are used to support RNA above them during this vulnerable period for the APOBEC. Notwithstanding this, we created multiple bindings of A3s and Vifs across species thousands of times to understand the repertoires of interactions. Moreover, our results in aggregate clearly show high similarity of structure through evolution across highly divergent proteins with intriguing binding similarities and variances, denoting regional and residue interactions on the surfaces of binding partners (Figure 9). Thus, the shifting of some of these interactions to RNA present during incorporation could provide a much stronger binding among all of the other residues seen across this study. This evolutionary arms race is also complicated by the slow lifespans of primates that use A3s as barriers to zoonosis, while viral Vif is able to consistently adapt from individual to individual. It could be said that once a zoonotic disease takes hold, the arms race is already partially lost; however, A3s may still remain a barrier against other zoonotic viruses attempting replication as well as retrotransposons [5,201]. A3 proteins restrict diverse DNA and RNA viruses, including some that may no longer exist [38]. Recent findings reveal a unique interaction between Epstein-Barr Virus (EBV) BORF2 and APOBEC3B across its DNA-binding groove, underscoring the complex evolutionary pressures shaping A3 diversification [202].

The structural conservation of APOBEC3 (A3) proteins, particularly A3F and A3G, across species reveals significant evolutionary adaptations in their DNA-binding mechanisms. CD1 and CD2 domains exhibit distinct properties, with CD1 showing highly positive charges and structural variability, while CD2 maintains a conserved DNA-binding groove. These differences suggest functional specialization, where CD1 facilitates interactions with DNA and other APOBEC molecules, enhancing synergistic antiviral effects, while CD2 supports DNA movement across the groove. One of the reasons A3F and A3G may have kept an inactive domain was to allow for greater movement across DNA by jumping from region to region [17,35,77,79,86,88,91,182]. Key structural motifs highlight evolutionary conservation and variability. The NRPIL motif in CD1, essential for dimerization through R24 and creating a positively charged surface, however, in both A3G-CD1 and A3F-CD1 among some primates, exists as a lysine or in NWMs and *Chlorocebus pygerythrus* as a glutamate [165,166]. A3F-CD2 regions exhibit highly negative patches (ℓ4’, α3’, α4’), contrasting with positively charged ℓ1’ residues over the catalytic pocket, supporting DNA binding. This charge distribution significantly affects DNA interactions. CD1 remains highly positive across taxa, while CD2 is more negative where charge dichotomy likely reflects an adaptive balance to facilitate DNA binding and catalysis. For instance, A3F-CD1 shows enhanced positive interactions attracting DNA phosphates, while A3G-CD1 employs hydrophobic residues in already polar regions, demonstrating domain-specific functional optimization.

The binding preference for specific DNA motifs reflects structural adaptations. A3F interacts predominantly with *TTC* motifs through hydrophobic π-π interactions, while A3G binds *CCC* motifs via polar and charged interactions between DNA bases and amino acids. Notably, the substrate specificity loop ℓ7’ (307YYFW310 in A3F; 315YDDQ318 in A3G) is critical for maintaining motif recognition and DNA binding. All the while, the 305RL306 of A3F and 313RI314 of A3G maintain the structure of this loop and connect it with ℓ1’. These conserved interactions underscore the importance of these loops in regulating enzymatic activity and substrate specificity. Across all species, ℓ1’, ℓ3’, ℓ5’, and ℓ7’ loops dominate DNA interactions, contributing over 75% of contacts in both proteins (Figure 10B,D and Figure S8, and Tables S23–S26) [80,82,88,89,90]. The loops form the DNA-binding groove with ℓ1’ shaping the U-like groove that stabilizes DNA binding and are critical for the efficiency of A3’s enzymatic mechanism. Specific residues, such as R213/R215 of A3F and A3G and the adjacent pocket gatekeeper positions (214/216), play structural roles by supporting the loop with the backbone of ℓ1’ and α1’ and enabling interactions with DNA backbones over the pocket, respectively [80,82,88,89,90,93,94,95,123,142,194,195,203,204,205]. Variations in these residues, such as asparagine in A3F and histidine in A3G, adapt domain functionality to distinct DNA-binding needs. The α-helices, though less involved than loops, provide critical structural support. For example, α4’ in A3F enables additional π-π interactions, stabilizing the sandwiching of π-π bonding in ℓ7’, while α6’ at position R374 in A3G interacts strongly with DNA phosphates, anchoring the DNA at the 5’ end. These helices complement the loops, enhancing DNA stabilization and catalytic efficiency.

APOBEC3 proteins exhibit extensive structural conservation, albeit positionally diverse, yet they retain flexibility to adapt to diverse functions. The linker region between domains, which is comprises eight residues in A3F and five residues in A3G, allows for dynamic conformational changes, as seen in Gorle et al. [91]. This flexibility enables catalytic pockets to adopt multiple orientations, facilitating diverse interactions with nucleotides, RNA, and reverse transcriptase. Homology models and previous studies reveal three major conformations: both catalytic pockets aligned, pockets in opposite orientations, or intermediate states [65,66,91,165,166,172]. These conformations impact DNA-binding efficiency and interactions. For example, double-domain APOBECs often position catalytic pockets near each other, creating a dual-binding interface for DNA or RNA. This configuration enhances interactions at key motifs, such as the 24RPIL28 region in A3G-CD1, which plays a role in virion packaging and catalysis [65,165,166]. Variations in this motif, such as glutamate or lysine adaptations in A3F of New World monkeys, and Maiti et al.’s substitution of R24A, revealed changes in RNA interactions occur in this region as well as reduced catalysis. The APOBECs during the uncoating and transcription phase of the lentiviral life cycle have the potential of interacting with reverse transcriptase, RNA, and DNA, which given double-domain APOBEC’s ability for dimerization and intersegmental transfer, means it may interact with multiple nucleotide chains or their regions at a time [63,165]. In the double-domain structures for which we have modelled 3D structural homology, we can see the possibility of a second route across the β-sheet of the CD1 domains, as the CD1 pocket and groove are blocked behind loop 3. While in this conformation, DNA could interact across this region which maintains similar structural surfaces and relative charges.

Docking studies further illustrate functional conservation despite structural variations. Preferred DNA motifs (5’-*taTTCat* for A3F and 5’-*ttCCCtt* for A3G) consistently position specific nucleotides within the catalytic pocket, particularly at position 4. Although docking algorithms differ in predicted nucleotide stability, the conserved binding patterns across species highlight the robustness of APOBEC-DNA interactions. However, it is also of note that due to the degrees of freedom that occur in DNA as compared to other small molecules, certain conformations may occur more often due to algorithmic scalability issues. Differences in catalytic efficiency between A3F and A3G align with these observations, with A3G displaying greater nucleotide and backbone interactions. The loops and helices collectively maintain the structural integrity and functionality of A3F and A3G. In ℓ7’, hydrophobic residues in A3F stabilize interactions with *TTC* motifs, while hydrophilic residues in A3G facilitate binding to *CCC* motifs. These substrate-specific adaptations illustrate the evolutionary tuning of APOBEC3 proteins to optimize their antiviral activities. Interactions at ℓ1’ further stabilize the DNA-binding groove, with positive residues in A3F enhancing phosphate interactions, and hydrophobic residues in A3G contributing to overall stabilization. Comparative analyses of α1’, α2’, and α3’ reveal their role in shaping the catalytic pocket and stabilizing DNA. For example, α1’ residues in A3F engage in hydrophobic and π-bonding interactions, while planar and polar residues in A3G strengthen DNA stabilization. α2’ and α3’ are integral in their shaping of the catalytic pocket. However, α4’ of A3F Y314 allowed for extra π-π bonding with the nucleotides stabilizing the sandwiching of π-π bonding in ℓ7’. These interactions are complemented by α6’ residues such as R374 in A3G, which anchor DNA through phosphate interactions, underscoring the helices’ supporting role in DNA binding. The observed structural variations across species highlight APOBEC3 proteins’ evolutionary adaptability while preserving critical functions. For example, the conformational flexibility enabled by the linker region allows APOBECs to interact with multiple nucleotide chains or different regions simultaneously, enhancing their antiviral efficacy. Structural models also suggest alternative DNA-binding routes when catalytic grooves are blocked, ensuring continued DNA processing. Despite structural shifts, conserved motifs, charge distributions, and substrate-binding loops demonstrate the evolutionary pressures to maintain functionality. This balance of flexibility and conservation enables APOBEC3 proteins to efficiently bind and process DNA, contributing to their role in antiviral defense.

## 5. Conclusions

Our study contributes significantly to the growing field of host–viral coevolution by providing insights into the evolutionary and structural dynamics of A3F, A3G, and Vif proteins. As the first research to construct extensive 3D structural homology models for these proteins across species, it offers a unique perspective on their sequential and three-dimensional evolution. By integrating structural modelling with ancestral sequence reconstruction (ASR), we uncover shifts in extinct proteins, broadening our understanding of the evolutionary arms race between restriction factors and their viral antagonists. The diversity observed in A3F and A3G, particularly in their sequence alignments and structural adaptations, reveals a clear evolutionary strategy. While CD1 exhibits greater sequence diversity than CD2, its higher positive charge facilitates binding nucleotide chains for intersegmental transfer and virion packaging. Despite this diversity, the DNA-binding grooves across taxa maintain structural and functional consistency, with conserved residues or their equivalents playing critical roles in DNA interactions. Notably, the greatest variation between A3F and A3G lies in ℓ7’, where hydrophobic and π-π interactions dominate in A3F, while A3G relies on polar and charged interactions. The substrate-binding grooves remain evolutionarily stable across taxa, suggesting an essential role in protein functionality across evolutionary lineages. Our findings underscore the importance of examining both structural and evolutionary aspects to understand the mechanisms driving host–virus dynamics. Future studies should expand sequence data and structural models for restriction factors, viral antagonists, and accessory proteins across more species. Investigating these proteins’ coevolution will enhance our knowledge of immune system development and inform predictions about viral adaptation and zoonotic risks. This work represents a novel approach to studying protein evolution by combining structural and sequence analyses, paving the way for deeper insights into coevolutionary processes and their implications for human health.

## Data Availability

The data that support the findings of this study are available from the corresponding author, Mani Larijani, upon reasonable request.

## Supplementary Materials

The following supporting information can be downloaded at: https://www.mdpi.com/article/10.3390/v17030393/s1, Figure S1: Amino acid sequence logos of Different HIV lineages; Figure S2: Identity and Similarity of Viral proteins; Figure S3: Identity and Similarity of Host proteins; Figure S4: A3G Full Alignment; Figure S5: Charged surfaces of Human A3FG and HIV Vif; Figure S6: Surface charge of Vif; Figure S7: Charges of A3FG across species and domains; Figure S8: A3FG and Vif CryoEM binding surfaces; Figure S9: Structures of ancestral sequence reconstruction; Figure S10: Dual-domain APOBEC3FG from humans and ASR; Figure S11: A3FGs host phylogeny; Figure S12: Viral Vif phylogenies; Table S1: Sequences of A3F across Species from NCBI or Ensemble; Table S2: Sequences of A3G across Species from NCBI; Table S3: Sequences of full Lentiviral genomes; Table S4: A3F-CD1 Models created through I-TASSER, Zn Coordinates, Templates, Confidence Scores and Ramachandran Plot information; Table S5: A3F-CD2 Models created through I-TASSER, Zn Coordinates, Templates, Confidence Scores and Ramachandran Plot information; Table S6: A3G-CD1 Models created through I-TASSER, Zn Coordinates, Templates, Confidence Scores and Ramachandran Plot information; Table S7: A3G-CD2 Models created through I-TASSER, Zn Coordinates, Templates, Confidence Scores and Ramachandran Plot information; Table S8: Ancestral A3F-CD1 Models created through I-TASSER, Zn Coordinates, Templates, Confidence Scores and Ramachandran Plot information; Table S9: Ancestral A3F-CD2 Models created through I-TASSER, Zn Coordinates, Templates, Confidence Scores and Ramachandran Plot information; Table S10: Ancestral A3G-CD1 Models created through I-TASSER, Zn Coordinates, Templates, Confidence Scores and Ramachandran Plot information; Table S11: Ancestral A3G-CD2 Models created through I-TASSER, Zn Coordinates, Templates, Confidence Scores and Ramachandran Plot information; Table S12: HIV/SIV Vif Models created through I-TASSER, Zn Coordinates, Templates, Confidence Scores and Ramachandran Plot information; Table S13: A3F-Identity and Similarity dependent on Most Likely Ancestral Sequence Reconstruction; Table S14: A3G-Identity and Similarity dependent on Most Likely Ancestral Sequence Reconstruction; Table S15: Interactions of A3F2 on Vif by secondary structure; Table S16: Interactions of Vif on A3F2 by secondary structure; Table S17: Interactions of A3G1 on Vif by secondary structure; Table S18: Interactions of Vif on A3G1 by secondary structure; Table S19: Interactions of A3F2 on Vif by Amino Acid; Table S20: Interactions of Vif on A3F2 by Amino Acid; Table S21: Interactions of A3G1 on Vif by Amino Acid; Table S22: Interactions of Vif on A3G1 by Amino Acid; Table S23: Overall interactions of A3F with *taTTCat* against different species across Secondary Structures; Table S24: Interactions across the species of A3F with individual Amino Acids; Table S25: Overall interactions of A3G with *ttCCCtt* against different species across Secondary Structures; Table S26: Interactions across the species of A3G with individual Amino Acids.

## Abbreviations

The following abbreviations are used in this manuscript:

APOBEC	Apolipoprotein B mRNA editing polypeptide like
A3F	APOBEC3F
A3G	APOBEC3G
Vif	Viral infectivity factor
GAs	Great apes
OWMs	Old World monkeys
NWMs	New World monkeys
