# Supplementary material for: Coevolution of Lentiviral Vif with Host A3F and A3G: Insights from Computational Modelling and Ancestral Sequence Reconstruction
Source: Viruses. 2025 Mar 10;17(3):393. doi: 10.3390/v17030393 (PMC11946711; doi:10.3390/v17030393)
Supplement: Supplementary file 1 [file viruses-17-00393-s001.zip › Supplemental Figures-Huebert-Larijani_Viruses-2024_APOBEC3FG-VIF-DNA.pdf]

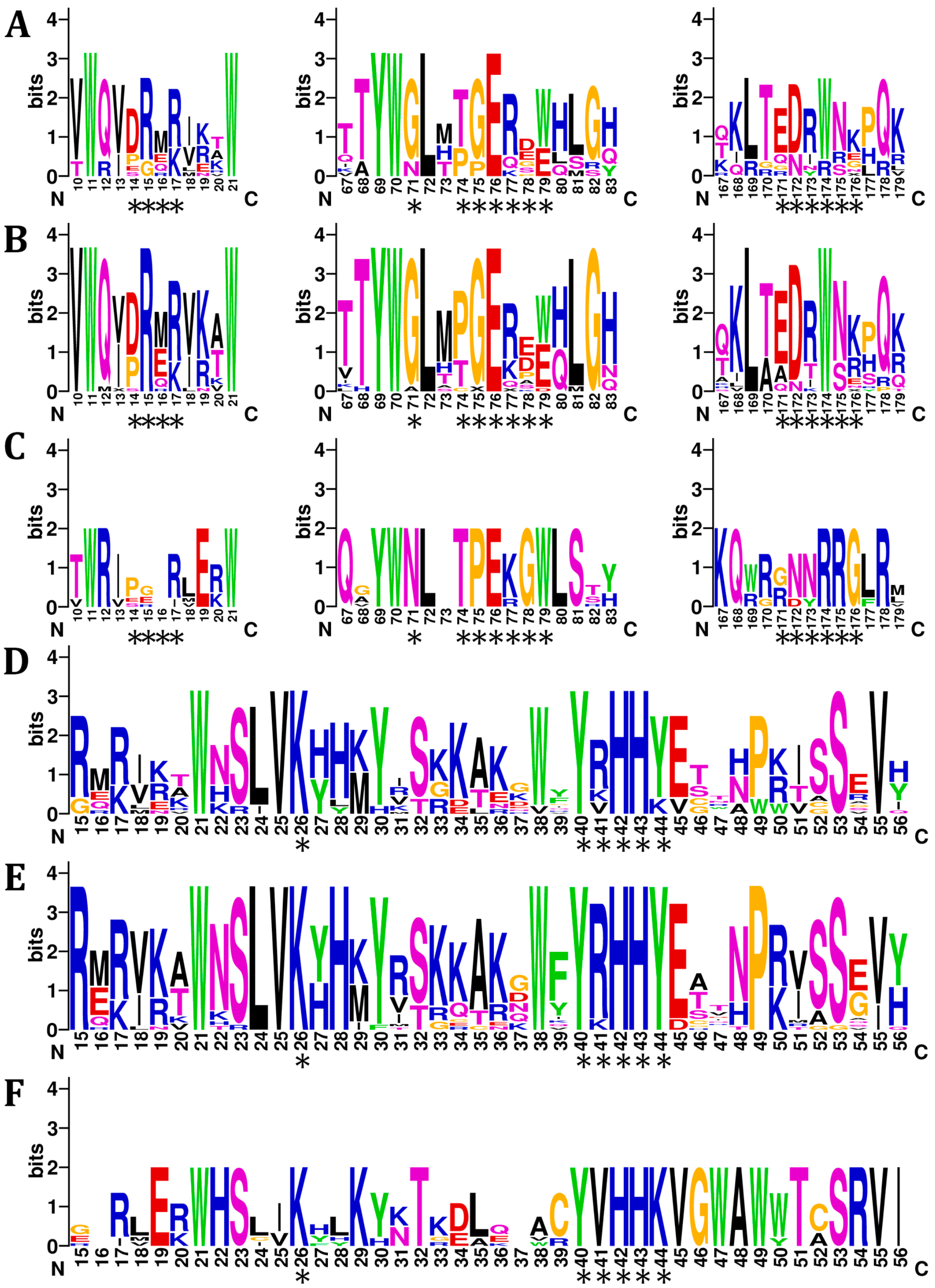

Figure S1: Amino acid sequence logos of Different HIV lineages.

All sequences are shown here with heights based on their confidence across alignments of all species across different proteins. Asterisks denote known residues that interact with their targets with Vif [330]. Vif sequences of binding sites of A3F (A-C) and A3G (D-F) are shown under 3 conditions: sequences (A & D), HIV-1 lineages including all HIV and SIV of Chimpanzee and Gorilla (B & E), and HIV-2 lineages including SIV of Sooty mangabey (C & F).

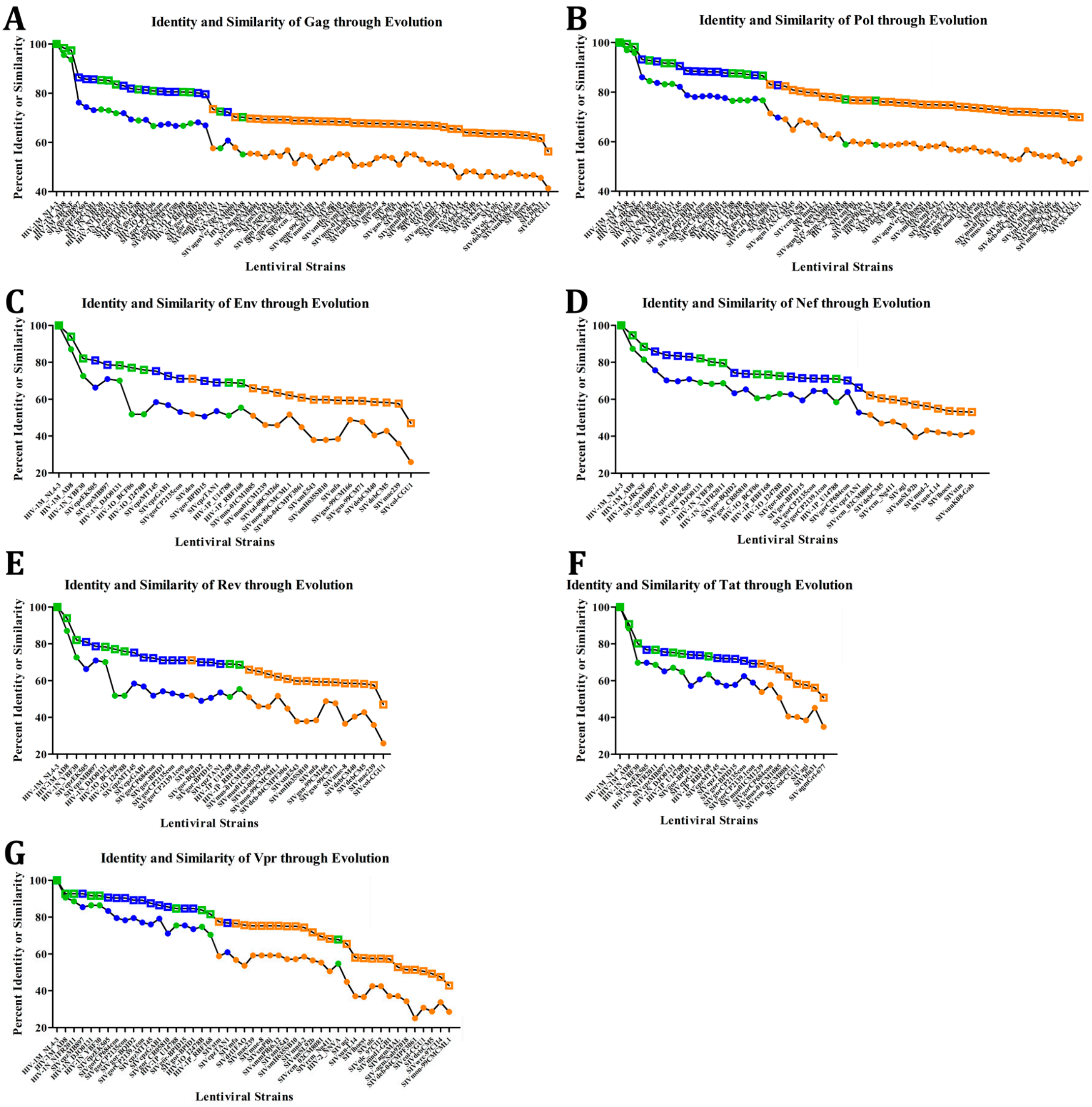

Figure S2: Identity and Similarity of Viral proteins.

The identity and similarity of viral structural proteins (A-C), and regulatory proteins (D-G) with colouring as per Figure 3.

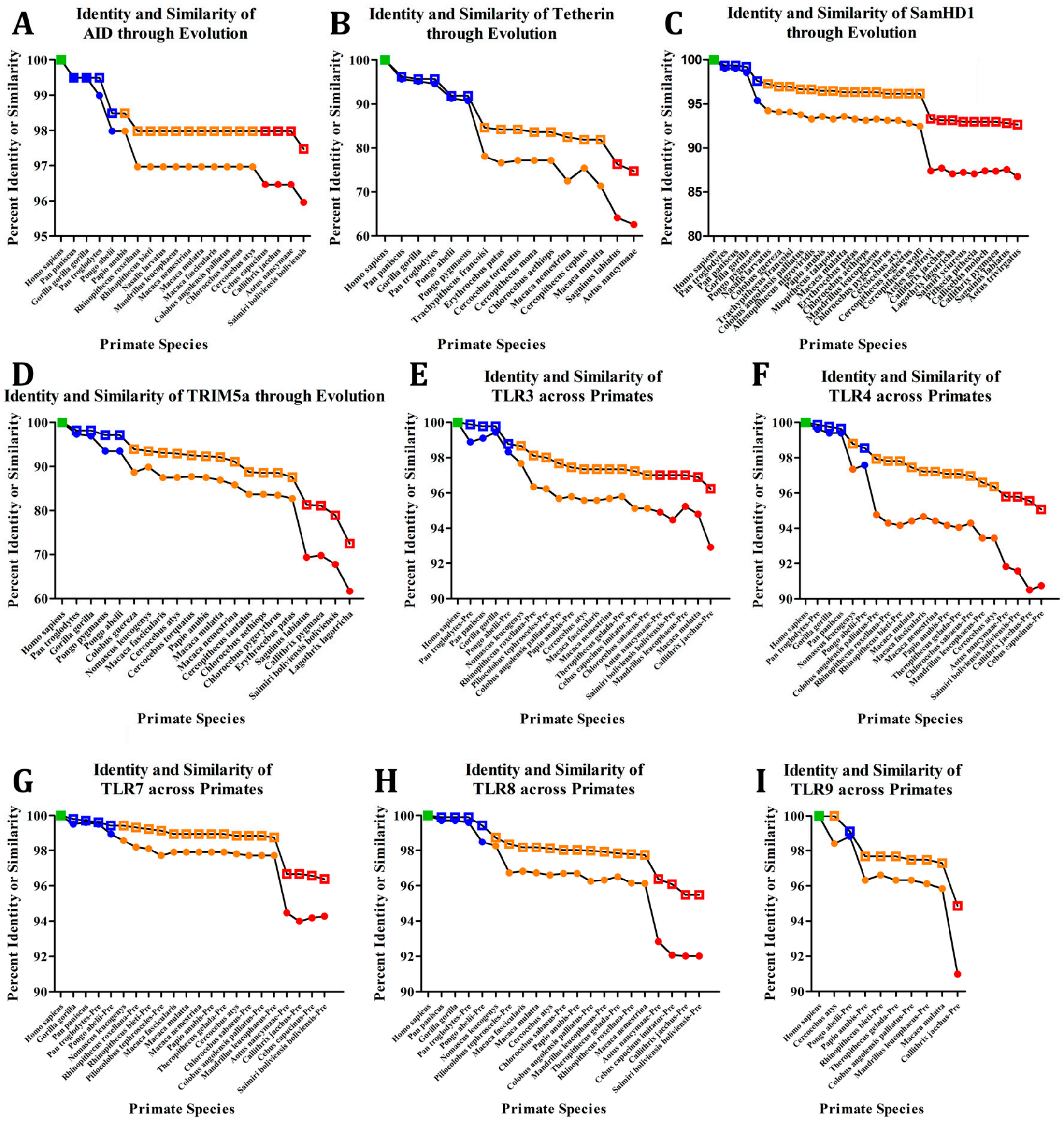

Figure S3: Identity and Similarity of Host proteins.

The identity and similarity of AID (A), other restriction factors (B-D), and toll-like receptors (E-I) with colouring as per Figure 3.

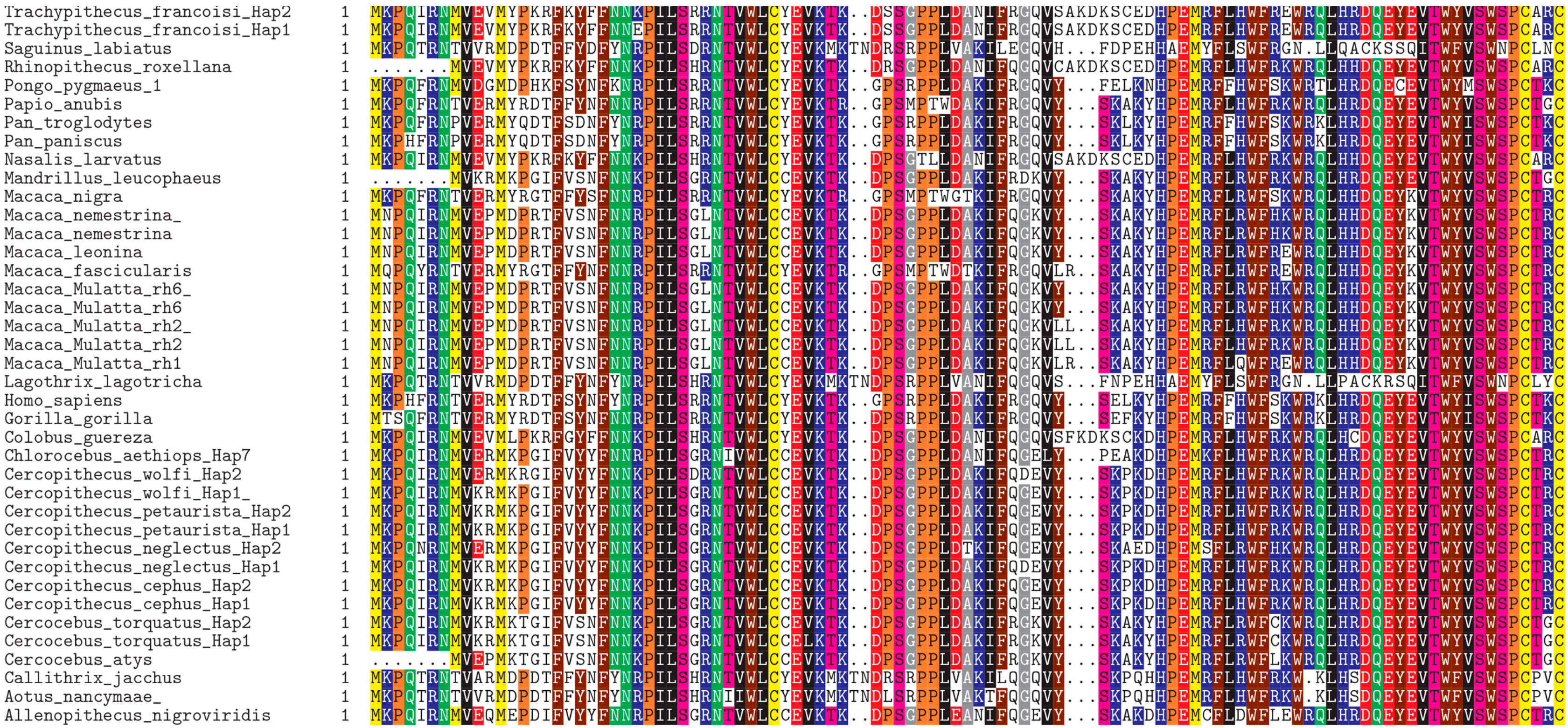

Figure S4: A3G Full Alignment (Part 1)

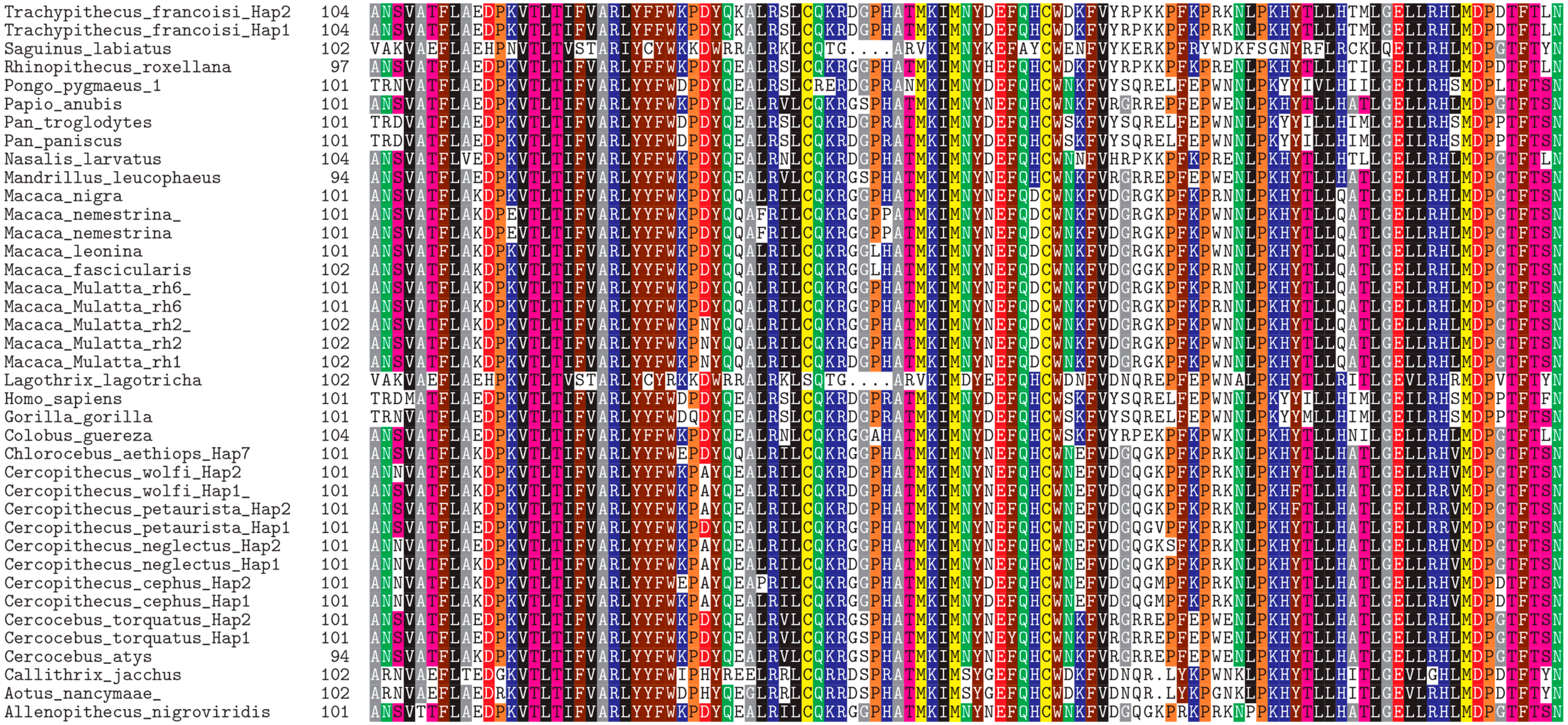

Figure S4: A3G Full Alignment (Part 2)

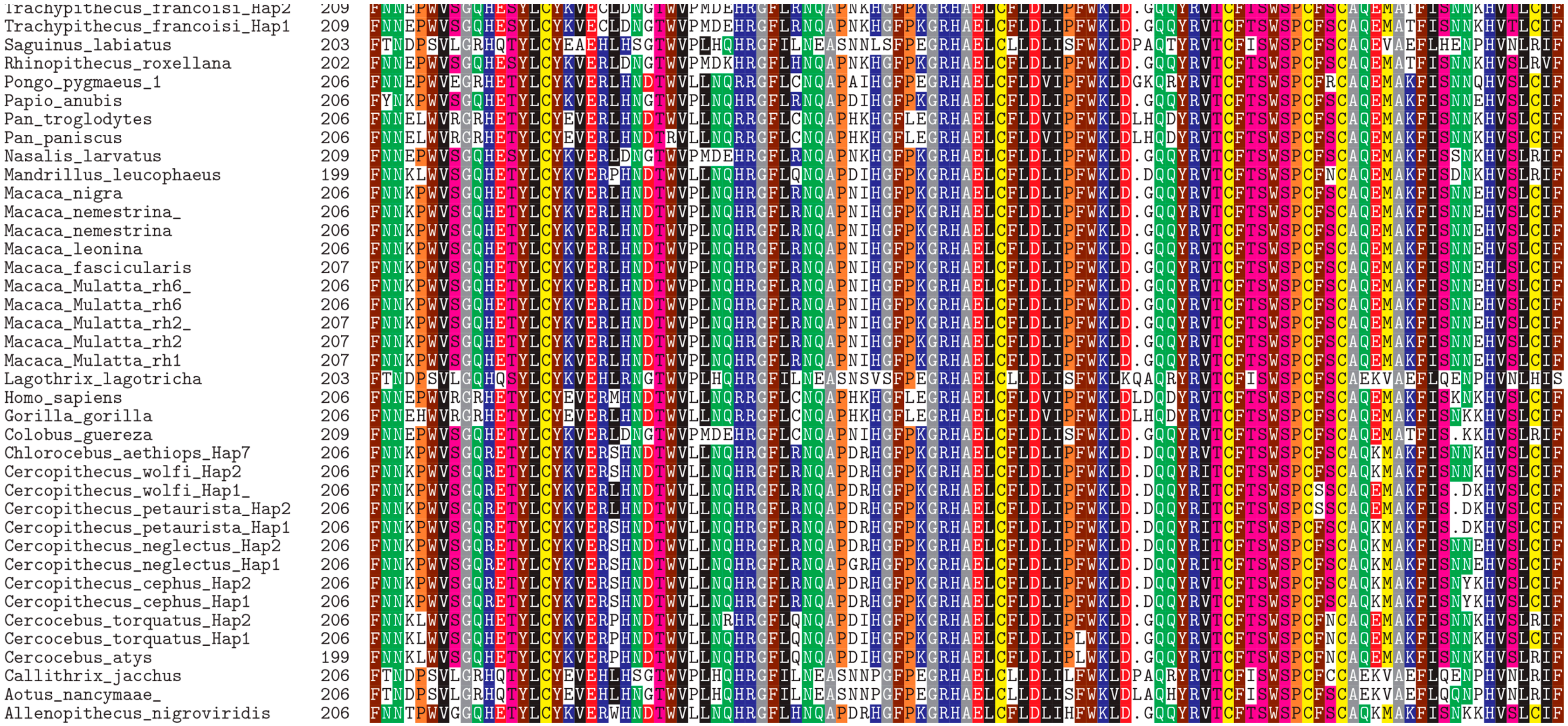

Figure S4: A3G Full Alignment (Part 3)

```
Trachypithecus_francoisi_Hap2    313  AARIYDDQGRGQEGLRALHTAGAEIAMMNYSEFKHCWDTFVDRQGRPFQPWDGLDEHSQALSGRLRAILQNQGN
Trachypithecus_francoisi_Hap1    313  AARIYDDQGRGQEGLRALHTAGAEIAMMNYSEFKHCWDTFVDRQGRPFQPWDGLDEHSQALSGRLRAILQNQGN
Saguinus_labiatus                308  AARIYDYRPGYEEGLLRLSWAGAPISMMKYSGFSHCWDTFVDHQGRSFKPWKGLNEHSQALSGRLQAILQIMGN
Rhinopithecus_roxellana          306  AARIYDDQGRGQEGLRALHTAGAEIAMMNYSEFKHCWDTFVDRQGRPFQPWDGLDEHSQALSGRLRAILQNQGN
Pongo_pygmaeus_1                 311  AARIYDDQGRCKEGLRTLDEAEAKISIMTYSEFQHCWDTFVDHQGRPFQPWDGLEEHSEAWSGKLQAILQNQGN
Papio_anubis                     310  AARIYDDQGRCQEGLRTLHRDGAKIAMMNYSEFEYCWDTFVDRQGRPFQPWDGLDEHSQDLSGRLRAILQNQGN
Pan_troglodytes                  311  AARIYDDQGRCQEGLRTLAKAGAKISIMTYSEFKHCWDTFVDHQGCPFQPWDGLEEHSQALSERLQAILQNQGN
Pan_paniscus                     311  AARIYDDQGRCQEGLRTLAKAGAEISIMTYSEFKHCWDTFVDHQGCPFQPWDGLEEHSQALSGRLRAILQNQGN
Nasalis_larvatus                 313  AARIYDDQGRGQEGLRALHTAGAEIAMMNYSEFKHCWDTFVDRQGRPFQPWDGLDEHSQALSGRLRAILQNQGN
Mandrillus_leucophaeus           303  AARIYDDQGRCQEGLRTLHRDGAKIAMMNYSEFEYCWDTFVDRQGRPFQPWDGLDEHSQDLSGRLRAILQNQGN
Macaca_nigra                     310  AARIYDDQGRYQEGLRTLHRDGAKIAMMNYSEFEYCWDTFVDRQGRPFQPWDGLDEHSQALSERLRAILQNQGN
Macaca_nemestrina_               310  AARIYDDQGRYQEGLRTLHRDGAKIAMMNYSEFEYCWDTFVDRQGRPFQPWDGLDEHSQALSGRLQAILQNQGN
Macaca_nemestrina                310  AARIYDDQGRYQEGLRTLHRDGAKIAMMNYSEFEYCWDTFVDRQGRPFQPWDGLDEHSQALSGRLQAILQNQGN
Macaca_leonina                   310  AARIYDDQGRYQEGLRTLHRDGAKIAMMNYSEFEYCWDTFVDRQGRPFQPWDGLDEHSQALSGRLRAILQNQGN
Macaca_fascicularis              311  AARIYDDQGRYQEGLRTLHRDGAKIAMMNYSEFKHCWDTFVDRQGRPFQPWDGLDEHSQALSERLRAILQNQGN
Macaca_Mulatta_rh6_              310  AARIYDDQGRYQEGLRALHRDGAKIAMMNYSEFEYCWDTFVDRQGRPFQPWDGLDEHSQALSGRLRAILQNQEN
Macaca_Mulatta_rh6               310  AARIYDDQGRYQEGLRALHRDGAKIAMMNYSEFEYCWDTFVDRQGRPFQPWDGLDEHSQALSGRLRAILQNQEN
Macaca_Mulatta_rh2_              311  AARIYDDQGRYQEGLRTLHRDGAKIAMMNYSEFEYCWDTFVDRQGRPFQPWDGLDEHSQALSERLRAILQNQGN
Macaca_Mulatta_rh2               311  AARIYDDQGRYQEGLRTLHRDGAKIAMMNYSEFEYCWDTFVDRQGRPFQPWDGLDEHSQALSERLRAILQNQGN
Macaca_Mulatta_rh1               311  AARIYDDQGRYQEGLRTLHRDGAKIAMMNYSEFEYCWDTFVDCQGCPFQPWDGLDEHSQALSERLRAILQNQGN
Lagothrix_lagotricha             308  AARIYDYQRGYKKGLRRLDRAGTPISMMKYSEFKHCWDTFVDHQGHPFQPWEELNEHSQALSGRLQAILQNQGN
Homo_sapiens                     311  TARIYDDQGRCQEGLRTLAEAGAKISIMTYSEFKHCWDTFVDHQGCPFQPWDGLDEHSQDLSGRLRAILQNQEN
Gorilla_gorilla                  311  AARIYDDQGRCQEGLRTLAEAGAKISIMTYSEFKHCWDTFVDHQGCPFQPWDGLEEHSQALSGRLQAILQNQGN
Colobus_guereza                  312  TARIYDDQGRCQEGLRTLHRVGAKIAMMNYSEFEYCWDTFVDHQGRPFQPWDGLDEHSQALSGRLRAILQNQGN
Chlorocebus_aethiops_Hap7        310  AARIYDDQGRCQEGLRTLHRDGAKIAVMNYSEFEYCWDTFVDRQGRPFQPWDGLDEHSQALSGRLRAILQNQGN
Cercopithecus_wolfi_Hap2         310  ATRIYDDQGRCQEGLRTLHRVGAKIAMMNYSEFEYCWDTFVDRQGRPFQPWDGLDEHSQALSGRLRAILQNQGN
Cercopithecus_wolfi_Hap1_        309  AARIYDDQGRCQEGLRTLHRDGAKIAMMNYSEFEYCWDTFVDHQGRPFQPWDGLDEHSQALSGRLRAILQNQGN
Cercopithecus_petaurista_Hap2    309  AARIYDDQGRCQEGLRTLHRDGAKIAMMNYSEFEYCWDTFVDHQGRPFQPWDGLDEHSQALSGRLRAILQNQGN
Cercopithecus_petaurista_Hap1    309  ATRIYDDQGRCQEGLRTLHRGGAKIAMMNYSEFEYCWDTFVDHQGRPFQPWDGLDEHSQALSGRLRAILQNQGN
Cercopithecus_neglectus_Hap2     310  AARIYDDQGRCQEGLRTLHRGGAKIAMMNYSEFEYCWDTFVDRQGHPFQPWDGLDEHSQALSGRLQAILQNQGN
Cercopithecus_neglectus_Hap1     310  AARIYDDQGRCQEGLRTLHRGGAKIAMMNYSEFEYCWDTFVDRQGHPFQPWDGLDEHSQALSGRLQAILQNQGN
Cercopithecus_cephus_Hap2        310  AARIYDDQGRCQEGLRTLHRGGAKIAMMNYSEFEYCWDTFVDRQGRPFQPWDGLDEHSQDLSGRLWAILQNQGN
Cercopithecus_cephus_Hap1        310  AARIYDDQGRCQEGLRTLHRGGAKIAMMNYSEFEYCWDTFVDRQGRPFQPWDGLDEHSQDLSGRLWAILQNQGN
Cercocebus_torquatus_Hap2        310  AARIYDDQGRCQEGLRTLHRDGAKIAMMNYSEFEYCWDTFVDRQGRPFQPWDGLDEHSQDLSERLRAILQNQGN
Cercocebus_torquatus_Hap1        310  AARIYDDQGRCQEGLRTLHRDGAKIAMMNYSEFEYCWDTFVDRQGRPFQPWDGLDEHSQDLSERLRAILQNQGN
Cercocebus_atys                  303  AARIYDDQGRCQEGLRTLHRDGAKIAMMNYSEFEYCWDTFVDRQGRPFQPWDGLDEHSQALSERLRAILQNQGN
Callithrix_jacchus               311  AARIYGYQRGYKKGLRRLNRAGAPISMMKYSEFSHCWDTFVDHHRCRFEPWEGLNEHSQALSGRLQAILQIMGN
Aotus_nancymaae_                 311  AARIYNYQRGYKKGLRRLDRAGAPISMMKYSEFRHCWDTFVDHQGHLFKPWEGLNEHSQALSGRLQAILQIMGN
Allenopithecus_nigroviridis      310  AARIYDDQGRCQEGLRTLHRVGAKIAMMNYSEFKHCWDTFVDRQGRPFQPWDGLDEHSQALSGRLRAILQNQGN
```

Figure S4: A3G Full Alignment (Part 4)

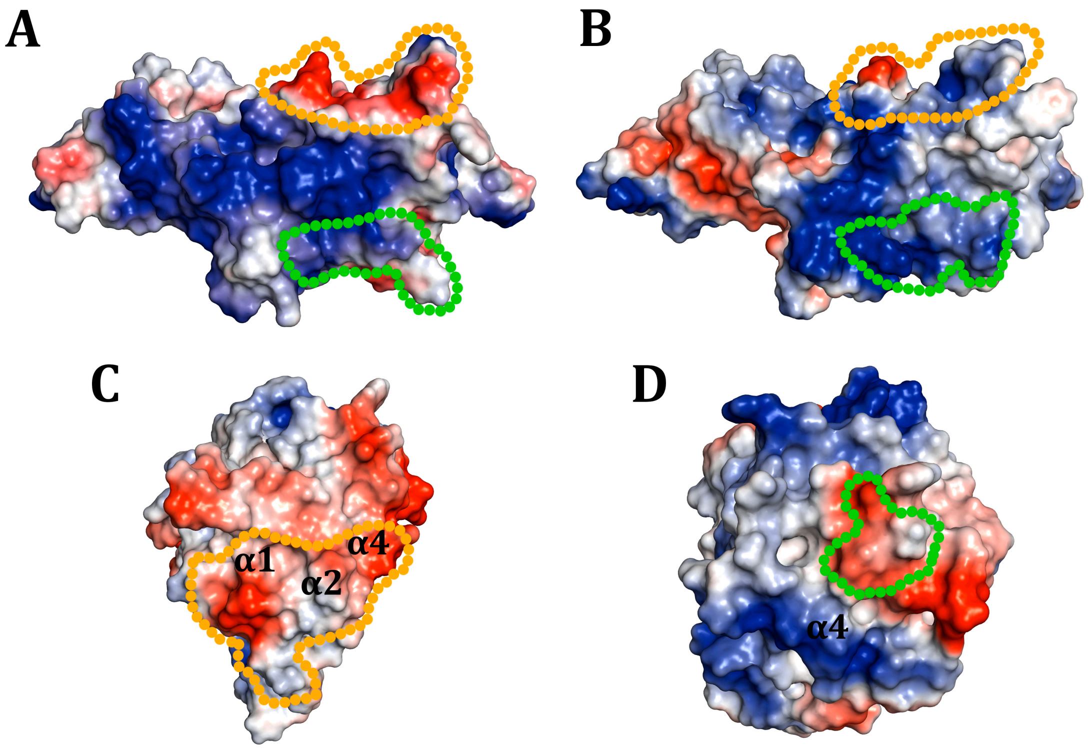

Figure S5: Charged surfaces of Human A3FG and HIV Vif.

Structures showing the vacuum electrostatics of HIV-1M NL4-3 (A), HIV-2 NNVA (B), A3F-CD2 (C), and A3G-CD1 (D) where blue denotes positively and red denotes negatively charged surfaces, and the orange dotted and green dotted lines are the binding sites between Vif and either A3G or A3F respectively.

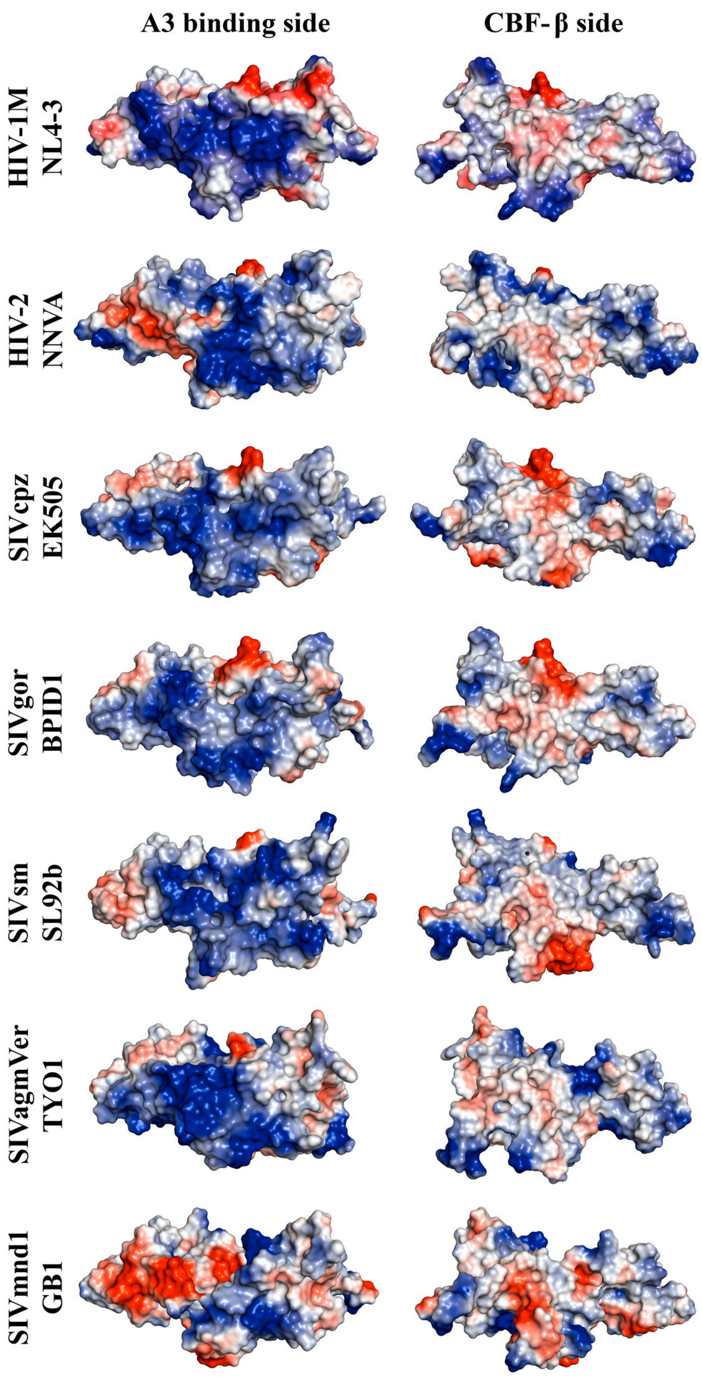

Figure S6: Surface charge of Vif.

Vacuum electrostatics are shown of HIV/SIV Vif denoting the side that binds A3s as well as the side that interact with CBF-β, where blue denotes positively and red denotes negatively charged surfaces.

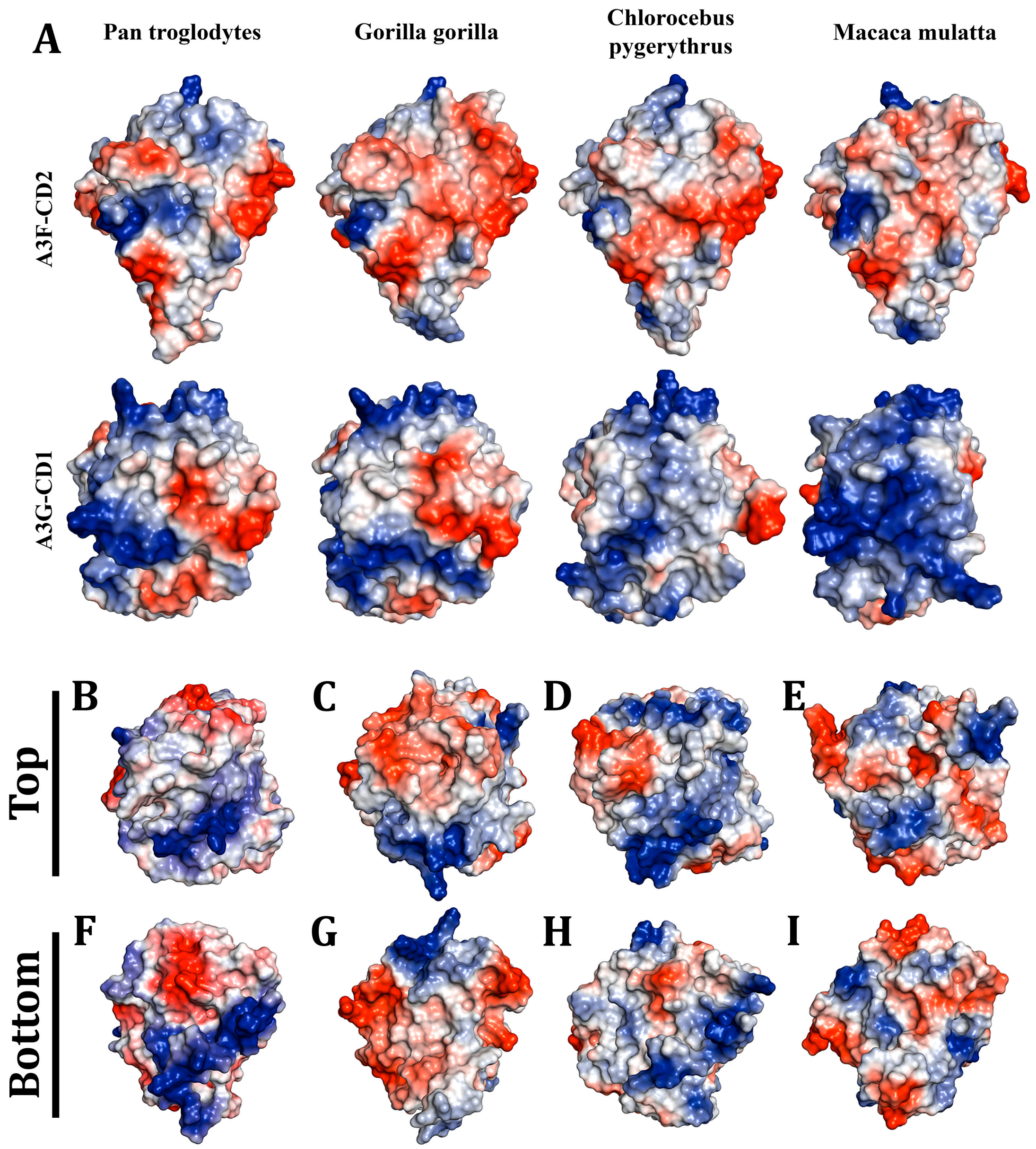

Figure S7: Charges of A3FG across species and domains.

Vacuum electrostatics are shown of A3F-CD2 and A3G-CD1 across primates (A), where blue denotes positive while red denotes negative charged surfaces. Angles shown are the same as in Figure 5. Charge surface structures of A3F-CD1 (B,F), A3F-CD2 (C,G), A3G-CD1 (D,H), and A3G-CD2 (E,I) shown from a top view of the DNA-binding groove (B,C,D,E) and from the β-sheet at the back of the protein (F,G,H,I).

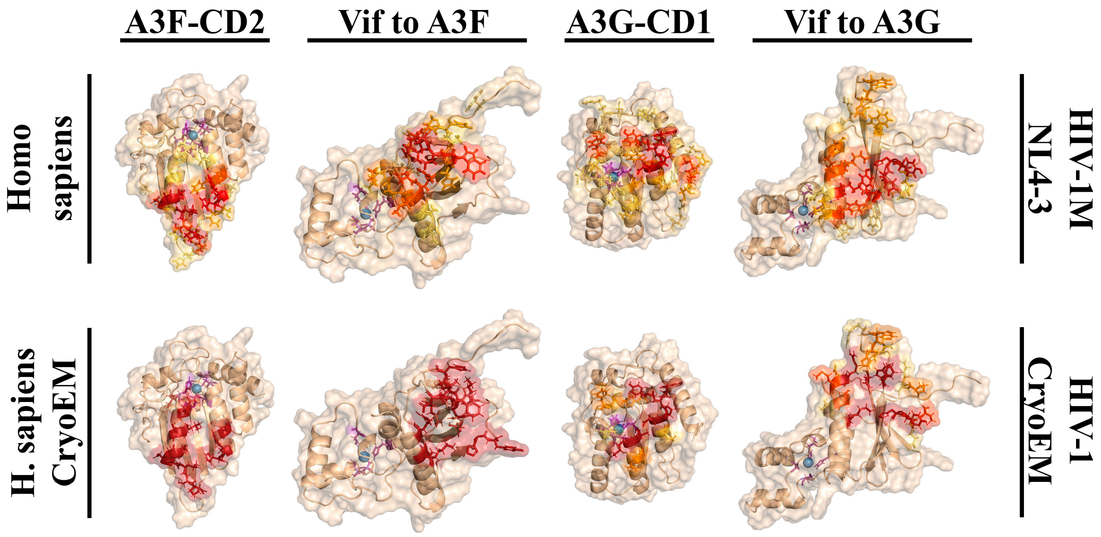

Figure S8: A3FG and Vif CryoEM binding surfaces.

Heatmaps of CryoEM interactions (overlayed on our I-TASSER structures) recorded when docking host A3FG is in contact with HIV/SIV Vif from CryoEM structures at the same angles as Figure 9. This data was analyzed in the same manner as the Haddock data with A3F-Vif data from 6NIL, and A3G-Vif data from 8J62, 8H0I, 8E40, 8CX0, 8CX1, and 8CX2. All 3D structural homology models within each column are shown at the same angle for ease of viewing interactions. Cartoons are shown with transparent surfaces, where zinc is shown as a sky-blue sphere; the HCCH motif of Vif and the catalytic residues of A3 are shown as purple sticks; and interactions are shown on a spectrum. The spectrum is shown based on percent interaction with wheat (0%), yellow-orange (25%), orange (50%), red (75%), and firebrick (100%). Sticks of residues were shown under the condition that the residues were interacted with at least 10% of the time.

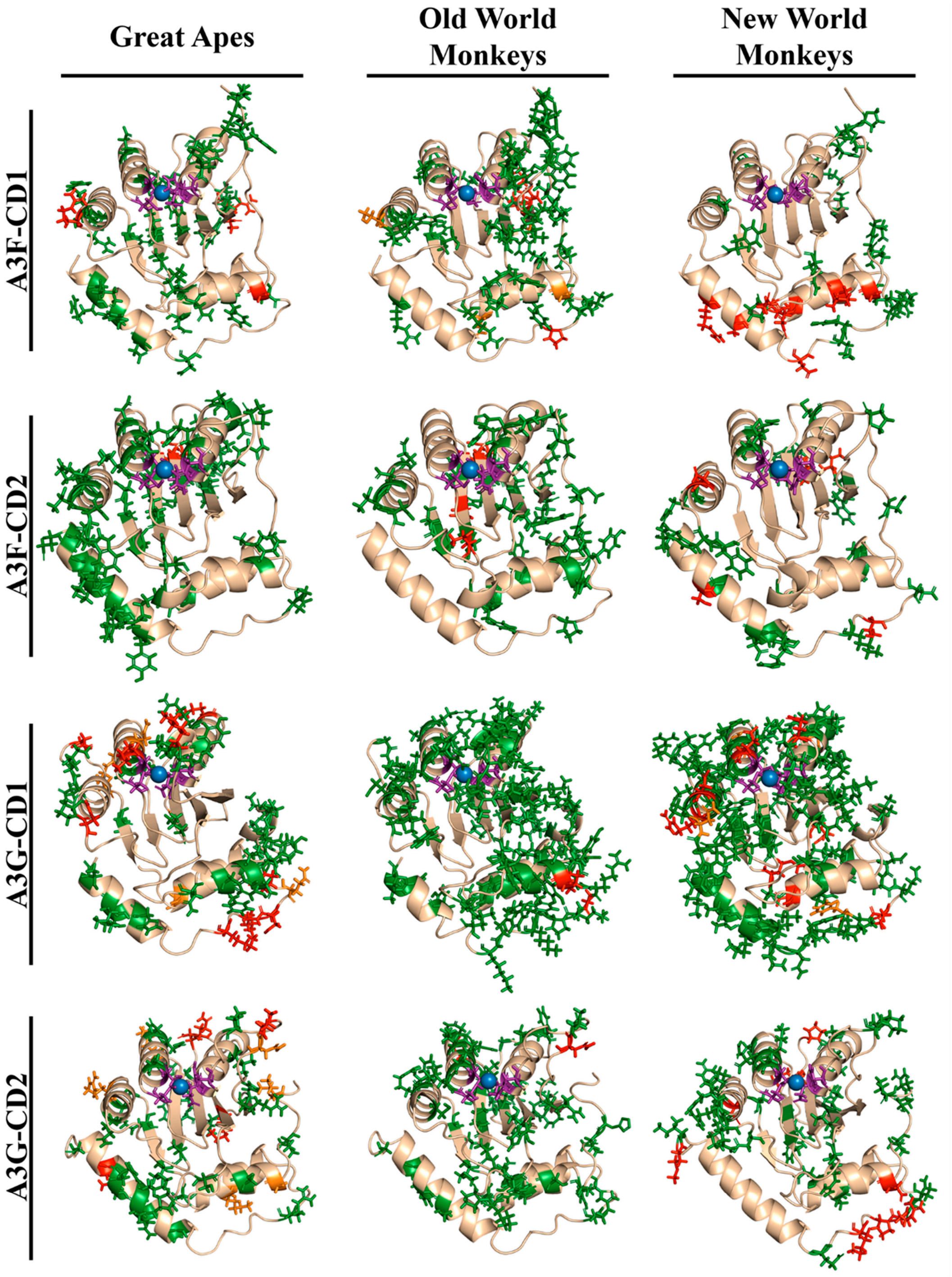

Figure S9: Structures of ancestral sequence reconstruction.

The most likely ancestral structures of A3FGs across taxa. Colours are shown as changes across taxa (green), residues that are uncertain (red), and uncertain residues which are highly likely with >90% certainty (orange).

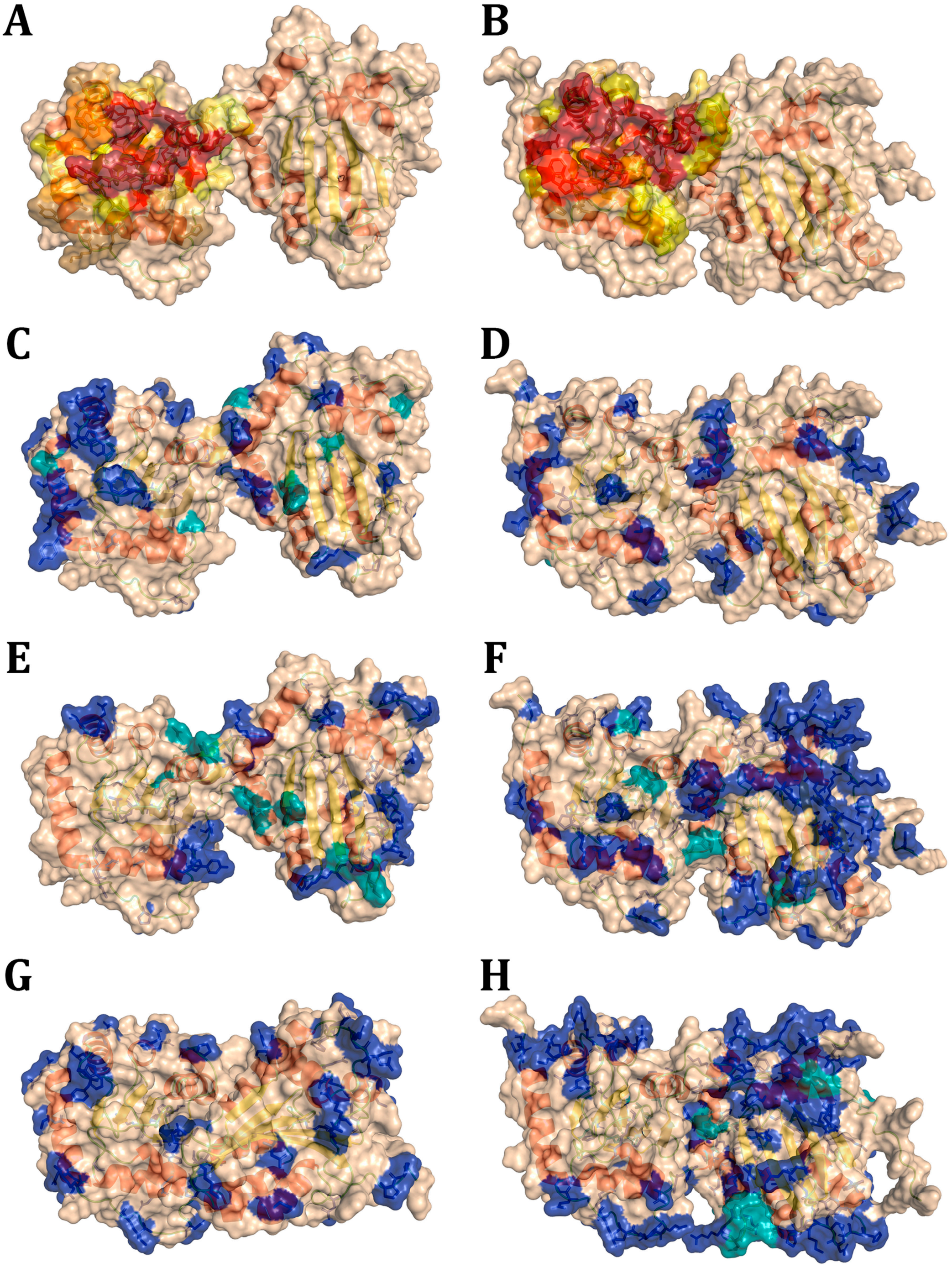

Figure S10: Dual-domain APOBEC3FG from humans and ASR.

Dual domain 3D structural homology models of *Homo sapiens* A3F (A) and A3G (B) shown with the heatmap colours of DNA interactions. ASR dual-domain 3D structural homology models of A3F (C, E, G) and A3G (D, F, H) across GA (C, D), OWM (E, F), and NWM (G, H). Colours show the variation across their own taxa compared to the extant species where blue is any adaptation to a different amino acid type, and teal is any adaptation to a different amino acid of the same type.

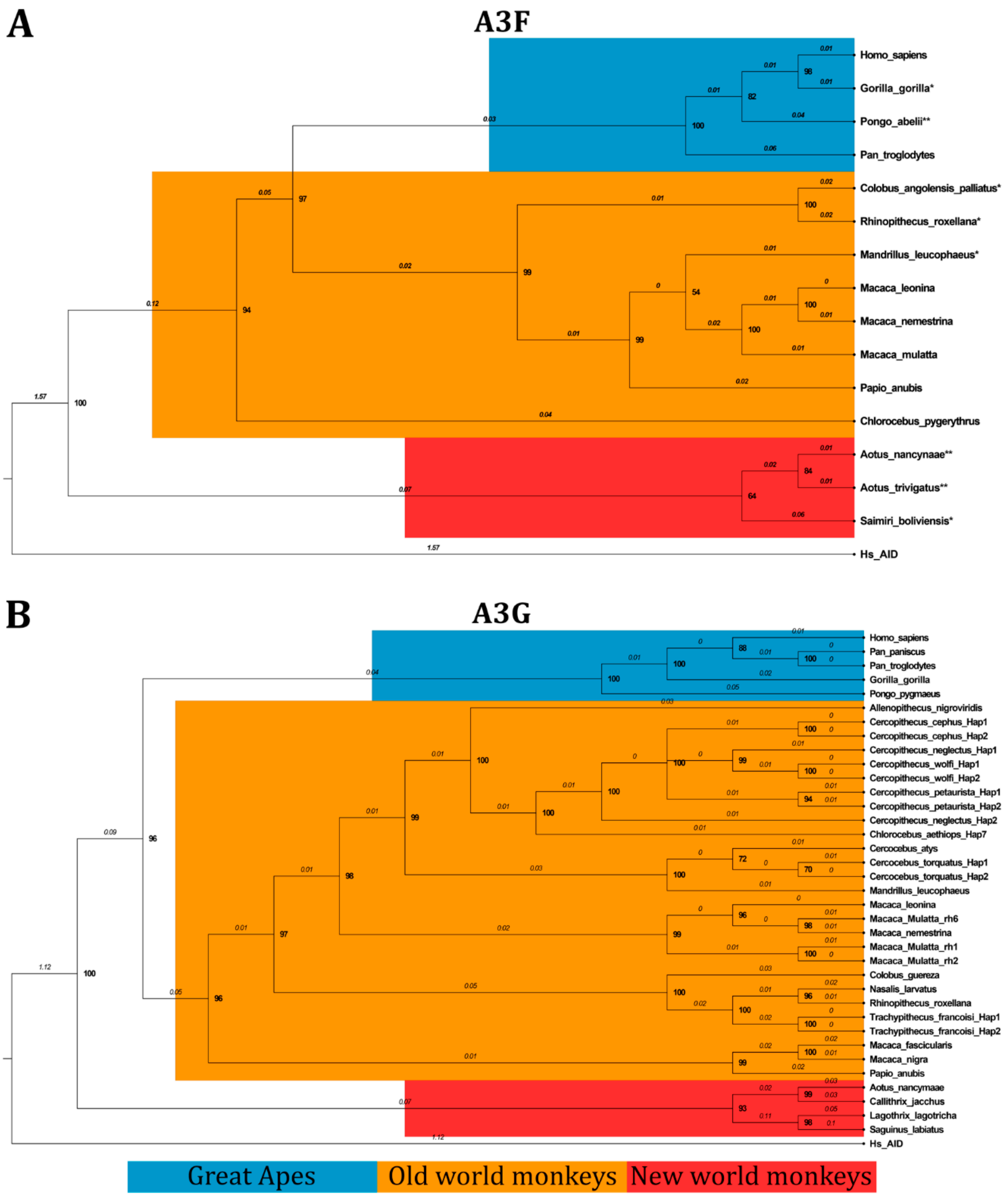

Figure S11: A3FGs host phylogeny.

These phylogenies denote the gene trees of A3F (A), and A3G (B). Colouration in both figures show GA (blue), OWM (orange), and NWM (red). [* denotes predicted mRNA transcript sequence, ** denotes sequences manually annotated from a larger genome]

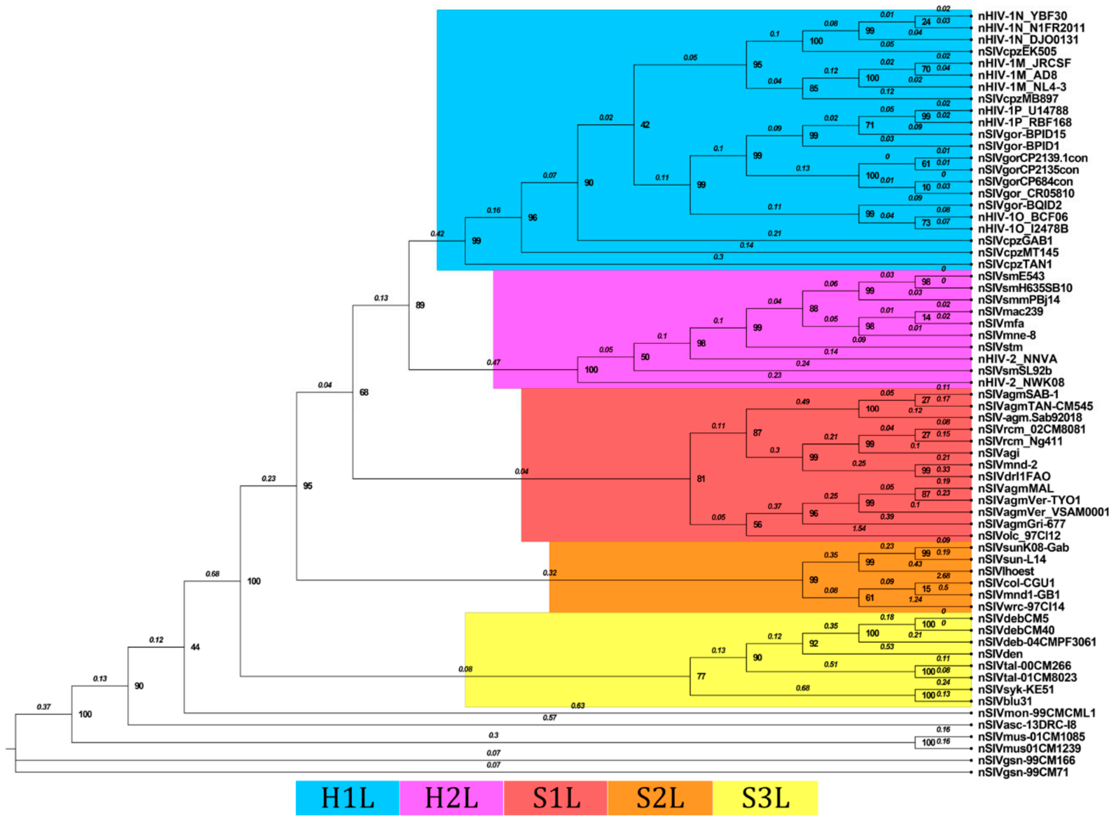

Figure S12: Viral Vif phylogenies.

This phylogeny is a gene tree of Vif. Colours denote 5 separate lineages: H1L (blue), H2L (purple), and 3 SIV lineages (S1L: red, S2L: orange and S3L: yellow).
